# Atomically Precise Ni–Pd Alloy Carbonyl Nanoclusters: Synthesis, Total Structure, Electrochemistry, Spectroelectrochemistry, and Electrochemical Impedance Spectroscopy

**DOI:** 10.1021/acs.inorgchem.1c02582

**Published:** 2021-10-21

**Authors:** Cristiana Cesari, Tiziana Funaioli, Beatrice Berti, Cristina Femoni, Maria Carmela Iapalucci, Federico Maria Vivaldi, Stefano Zacchini

**Affiliations:** † Dipartimento di Chimica Industriale “Toso Montanari”, Università di Bologna, Viale Risorgimento 4, 40136 Bologna, Italy; ‡ Dipartimento di Chimica e Chimica Industriale, Università di Pisa, Via G. Moruzzi 13, 56124 Pisa, Italy

## Abstract

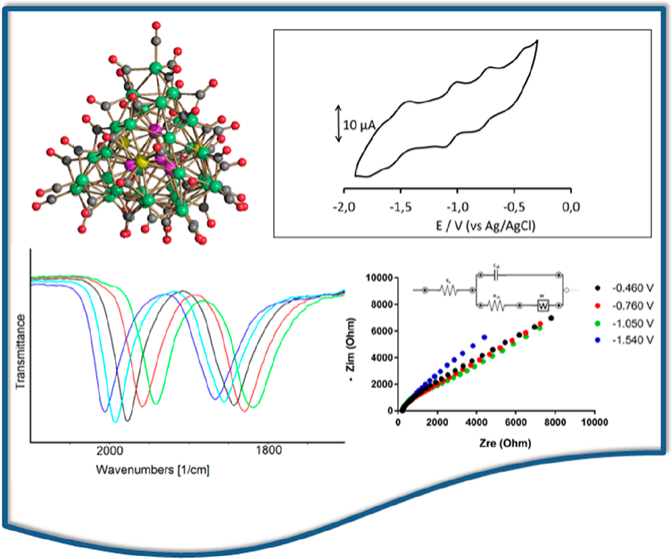

The molecular nanocluster [Ni_36–*x*_Pd_5+*x*_(CO)_46_]^6–^ (*x* = 0.41) (**1**^**6–**^) was obtained from the reaction of [NMe_3_(CH_2_Ph)]_2_[Ni_6_(CO)_12_] with 0.8 molar equivalent of [Pd(CH_3_CN)_4_][BF_4_]_2_ in tetrahydrofuran (thf). In contrast, [Ni_37–*x*_Pd_7+*x*_(CO)_48_]^6–^ (*x* = 0.69) (**2**^**6–**^) and [HNi_37–*x*_Pd_7+*x*_(CO)_48_]^5–^ (*x* = 0.53) (**3**^**5–**^) were obtained from the reactions of [NBu_4_]_2_[Ni_6_(CO)_12_] with 0.9–1.0 molar equivalent of [Pd(CH_3_CN)_4_][BF_4_]_2_ in thf. After workup, **3**^**5–**^ was extracted in acetone, whereas **2**^**6–**^ was soluble in CH_3_CN. The total structures of **1**^**6–**^, **2**^**6–**^, and **3**^**5–**^ were determined with atomic precision by single-crystal X-ray diffraction. Their metal cores adopted cubic close packed structures and displayed both substitutional and compositional disorder, in light of the fact that some positions could be occupied by either Ni or Pd. The redox behavior of these new Ni–Pd molecular alloy nanoclusters was investigated by cyclic voltammetry and in situ infrared spectroelectrochemistry. All three compounds **1**^**6–**^, **2**^**6–**^, and **3**^**5–**^ displayed several reversible redox processes and behaved as electron sinks and molecular nanocapacitors. Moreover, to gain insight into the factors that affect the current–potential profiles, cyclic voltammograms were recorded at both Pt and glassy carbon working electrodes and electrochemical impedance spectroscopy experiments performed for the first time on molecular carbonyl nanoclusters.

## Abstract 2

The synthesis, molecular structure, electrochemical, and infrared spectroelectrochemical behavior of three new atomically precise Ni−Pd alloy carbonyl nanoclusters are reported, together with the first electrochemical impedance spectroscopy investigation of redox active carbonyl nanoclusters.

## 1 Introduction

Very often, high-nuclearity metal carbonyl clusters (HNMCCs) display extended redox activity affording reversible electron cascades.^1−5^ In this sense, they are multivalent and display electron-sink behavior; that is, they are able to accept and release electrons reversibly in sequence at well-defined potentials. This behavior is somehow related to the fact that the metallic core of the cluster undergoes a transition from an insulator to a semiconductor and, eventually, conductor regime as its size (nuclearity) increases. As this metal core is shielded by an insulator layer of carbon monoxide, multivalent HNMCCs may be viewed as molecular nanocapacitors.^1,2^

This electron-sink behavior enables the application of metal carbonyl clusters in electrocatalytic processes.^1^ In particular, molecular Fe and Co carbonyl clusters have been employed as electrocatalysts for the two-electron reduction of two protons to molecular hydrogen and for the two-electron reduction of H^+^ and CO_2_ to formate.^6,7^ The molecular nature of these clusters allows us to gain detailed structural insight into the electrocatalytic active species and into the overall mechanism occurring in the catalyst microenvironment.^8^ In this sense, the study of larger and larger molecular clusters such as HNMCCs represents an exciting frontier that could create a bridge to contemporary nanomaterials for electronic and electrochemical applications.^9−13^

The redox behavior of HNMCCs has been investigated by electrochemical and spectroelectrochemical methods, and recently, we reported the first two cases of multivalence in large Ni–Pd molecular nanoclusters.^14^ In several cases, in situ infrared spectroelectrochemistry (IR SEC) proved to be crucial in proving the existence of different stable redox states in HNMCCs.^15−18^ This is due to the fact that often low currents and broad peaks are observed in the current–potential profile from cyclic voltammetry (CV) of large clusters, and in several cases, the peaks are hardly observable. Therefore, the chemical and electrochemical reversibility of the redox processes, as well as the number of exchanged electrons, cannot be inferred from CV and controlled potential coulometric measurements.

When the redox chemistry of a carbonyl complex is studied by IR SEC in an optically transparent thin layer electrochemical (OTTLE) cell,^19^ the potential of the working electrode (WE) is swept at a slow rate between selected potentials and a sequence of vibrational spectra in the CO stretching region (ν_CO_) is collected at constant time intervals. Via proper selection of the cyclic potential range, it is possible to observe a completely reversible shift of the ν_CO_ bands of the complexes toward higher or lower wavenumbers as a consequence of an oxidation or a reduction, respectively, according to the scan direction. This applies to both terminal (ν^t^_CO_) and bridging (ν^b^_CO_) carbonyl groups.

Multiple redox changes occur sweeping the WE potential on solutions of multivalent HNMCCs. The complete sequence of collected IR spectra can be separated into groups each belonging to a different redox step, and the spectra assigned to the electrogenerated long-lived species with different charges. Moreover, the presence or absence of well-defined isosbestic points in each group of spectra indicates the relative stability of the electrogenerated species or other phenomena complicating the electron transfers, while a nearly uniform shift of the ν^t^_CO_ bands within a range of 14–20 cm^–1^ appears to be consistent with a one-electron step for HNMCCs.^20−22^ Accordingly, a double shift of ∼28 cm^–1^ was observed for bielectronic processes.^18,23^

Two further interesting pieces of information can be gathered from the IR SEC analysis: (a) the stability of the electrogenerated species on a time scale greater than that of CV, eventually suggesting the possibility of synthesizing and isolating such reduced or oxidized species, and (b) the structural variations ensuing from the redox processes that cause an appreciable modification of the IR spectra. For example, in several instances, we found that changing the cluster charge resulted in a variation in the relative intensity of terminal and bridging ν_CO_ bands, indicating structural changes with regard to the stereochemistry of CO ligands,^15^ according to the more favored bridging coordination mode for CO ligands at increased negative charges on a cluster.^24,25^

From a more general perspective, atomically precise metal nanoclusters and alloy nanoclusters have attracted renewed interest in recent years.^26−39^ Electrochemistry has been extremely powerful in investigating the electronic structure of thiolate-protected nanoclusters as well as unraveling doping (alloying) effects.^40−42^ Heterometallic HNMCCs may be viewed as atomically precise alloy nanoclusters and may contribute to a better understanding of the effects of size and structure on molecular clusters.^1−3,43,44^ In particular, we were interested in shedding more light on the different phenomena affecting their electrochemical behavior. Herein, we report the synthesis of three new heterometallic HNMCCs, [Ni_36–*x*_Pd_5+*x*_(CO)_46_]^6–^ (*x* = 0.41) (**1**^**6–**^), [Ni_37–*x*_Pd_7+*x*_(CO)_48_]^6–^ (*x* = 0.69) (**2**^**6–**^), and [HNi_37–*x*_Pd_7+*x*_(CO)_48_]^5–^ (*x* = 0.53) (**3**^**5–**^). Their total structures have been determined by single-crystal X-ray diffraction (SC-XRD), and their redox behavior has been investigated by CV and in situ IR SEC. Moreover, to gain insight into the factors that affect the current–potential profiles, the cyclic voltammograms were recorded at both Pt and glassy carbon (GC) working electrodes and electrochemical impedance spectroscopy (EIS) experiments performed for the first time on molecular carbonyl nanoclusters.

## 2 Results and Discussion

### 2.1 Synthesis and Molecular Structures

**Scheme 1:**
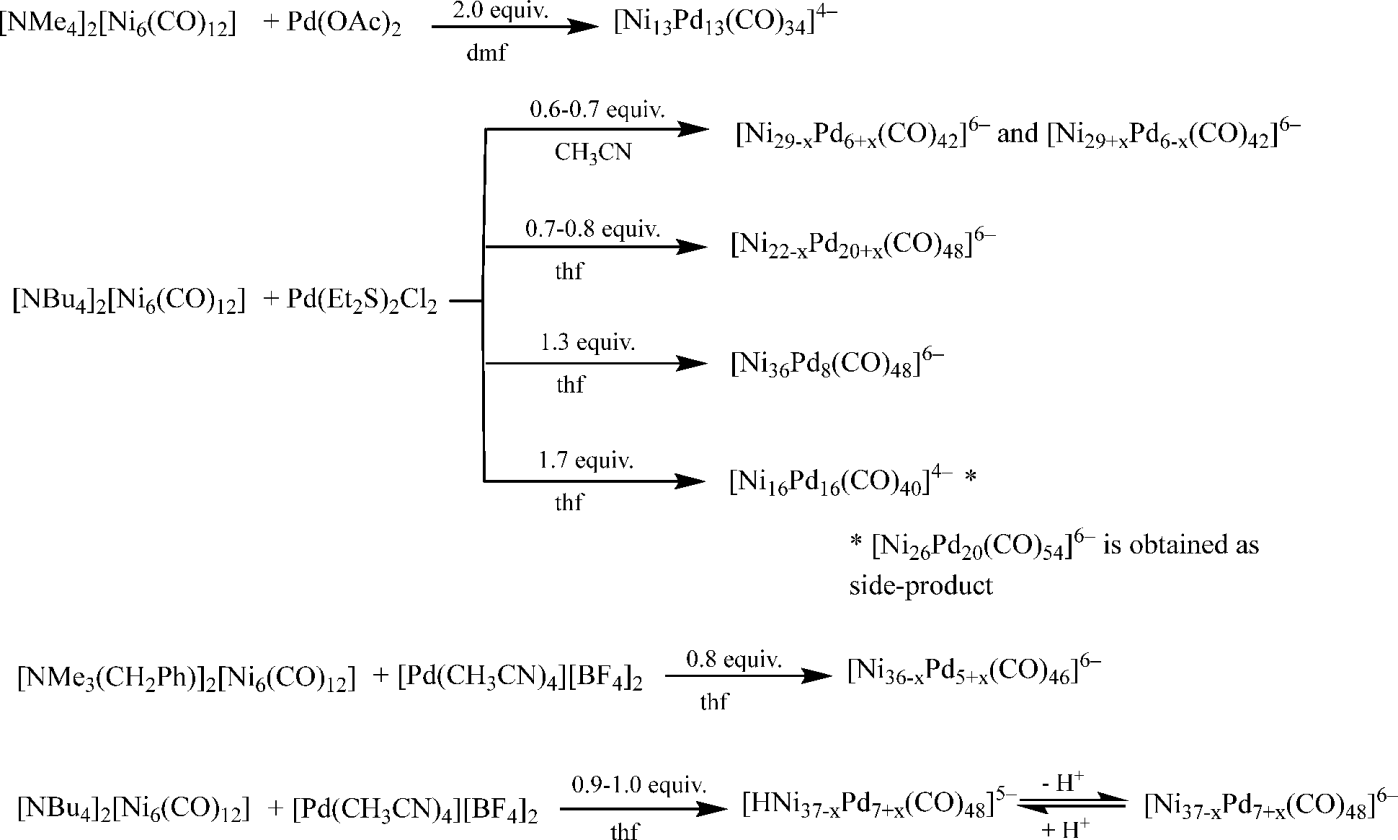
Synthesis of Ni–Pd–CO Clusters

Bimetallic Ni–Pd carbonyl clusters can be conveniently obtained from the redox condensation of [Ni_6_(CO)_12_]^2–^ with miscellaneous Pd(II) salts (Scheme 1).^1,14^ The reaction between [Ni_6_(CO)_12_]^2–^ and Pd(II) salts is strongly affected by several parameters such as (a) the [Ni_6_(CO)_12_]^2–^ cation, (b) the ligands coordinated to Pd(II), (c) the solvent, (d) the stoichiometric ratio of the reagents, (e) the rate of the addition of Pd(II) to [Ni_6_(CO)_12_]^2–^, and (f) whether CO is removed during the reaction. By carefully controlling all of these parameters, one can obtain several high-nuclearity Ni–Pd carbonyl clusters; [Ni_36_Pd_8_(CO)_48_]^6–^,^45^ [Ni_13_Pd_13_(CO)_34_]^4–^,^46^ [Ni_16_Pd_16_(CO)_40_]^4–^,^47^ and [Ni_26_Pd_20_(CO)_54_]^6–^^47^ have already been fully characterized. More recently, we have reported the synthesis and total structural characterization of [Ni_22–*x*_Pd_20+*x*_(CO)_48_]^6–^ (*x* = 0.63), [Ni_29–*x*_Pd_6+*x*_(CO)_42_]^6–^ (*x* = 0.09), and [Ni_29+*x*_Pd_6–*x*_(CO)_42_]^6–^ (*x* = 0.27).^14^ [Ni_22–*x*_Pd_20+*x*_(CO)_48_]^6–^ (*x* = 0.63) has been obtained from the reaction of [NBu_4_]_2_[Ni_6_(CO)_12_] with 0.7–0.8 molar equivalent of Pd(Et_2_S)_2_Cl_2_ in CH_2_Cl_2_, whereas [Ni_29–*x*_Pd_6+*x*_(CO)_42_]^6–^ (*x* = 0.09) and [Ni_29+*x*_Pd_6–*x*_(CO)_42_]^6–^ (*x* = 0.27) have been obtained from the reaction of [NEt_4_]_2_[Ni_6_(CO)_12_] with 0.6–0.7 molar equivalent of Pd(Et_2_S)_2_Cl_2_ in CH_3_CN.^14^

During this work, we have determined the following conditions for the syntheses of the new species [Ni_36–*x*_Pd_5+*x*_(CO)_46_]^6–^ (*x* = 0.41) (**1**^**6–**^), [Ni_37–*x*_Pd_7+*x*_(CO)_48_]^6–^ (*x* = 0.69) (**2**^**6–**^), and [HNi_37–*x*_Pd_7+*x*_(CO)_48_]^5–^ (*x* = 0.53) (**3**^**5–**^). In all of these reactions, CO was periodically removed under reduced pressure and the Pd(II) reagent was added in small portions.

(a) **1**^**6–**^ has been obtained by reacting [NMe_3_(CH_2_Ph)]_2_[Ni_6_(CO)_12_] with 0.8 molar equivalent of [Pd(CH_3_CN)_4_][BF_4_]_2_ in tetrahydrofuran (thf), and the final product extracted in CH_3_CN after workup.

(b) **2**^**6–**^ and **3**^**5–**^ have been obtained from the reactions of [NBu_4_]_2_[Ni_6_(CO)_12_] with 0.9–1.0 molar equivalent of [Pd(CH_3_CN)_4_][BF_4_]_2_ in thf. After workup, **3**^**5–**^ was extracted in acetone, whereas **2**^**6–**^ was soluble in CH_3_CN. **2**^**6–**^ and **3**^**5–**^ can be interconverted by means of acid–base reactions.

**1**^**6–**^, **2**^**6–**^, and **3**^**5–**^ have been structurally characterized by SC-XRD as their [NMe_4_]_2_[NMe_3_CH_2_Ph]_4_[**1**]·3CH_3_CN·solv, [NBu_4_]_6_[**2**]·6CH_3_CN, and [NBu_4_]_5_[**3**]·2CH_3_COCH_3_·solv salts, respectively. Their molecular structures are discussed below.

The presence of one hydride ligand on **3**^**5–**^ has been indirectly inferred from the fact that it is quantitatively deprotonated to the related hexa-anion by strong bases such as NaOH, and the reaction is completely reversed after the addition of strong acids such as HBF_4_·Et_2_O, as evidenced by IR spectroscopy. Similarly, **2**^**6–**^ can be protonated to the related penta-anion with HBF_4_·Et_2_O, and the reaction is reversed using NaOH. The fact that these penta/hexa-anion couples are reversibly interconverted by acid–base reactions suggests that these are protonation/deprotonation and not redox reactions. Moreover, **2**^**6–**^ and **3**^**5–**^ have very similar metal compositions (Ni_36.31_Pd_7.69_ and Ni_36.47_Pd_7.53_, respectively). Thus, the fact that they display different electrochemical properties (see section 2.2) is better explained assuming that **3**^**5–**^ is a hydride and **2**^**6–**^ is not, rather than looking at the Ni and Pd contents, which are almost identical. Moreover, similar protonation/deprotonation reactions have been previously reported for the related [Ni_36_Pd_8_(CO)_48_]^6–^ and [Ni_35_Pt_9_(CO)_48_]^6–^ clusters.^45^ The problem of detecting hydrides in HNMCCs has been previously discussed in the literature, and it has been shown that above a nuclearity of 20–25 metal atoms, the hydride resonances in the ^1^H nuclear magnetic resonance (NMR) spectra become very broad and eventually HNMCCs become NMR silent.^3,48^ In agreement with this trend, it has not been possible to directly detect the hydride ligand of **3**^**5–**^ by ^1^H NMR spectroscopy under any experimental condition employed (concentration, solvent, temperature, or field). Thus, we can only indirectly infer its hydride nature on the basis of the considerations reported above.

**Figure 1:**
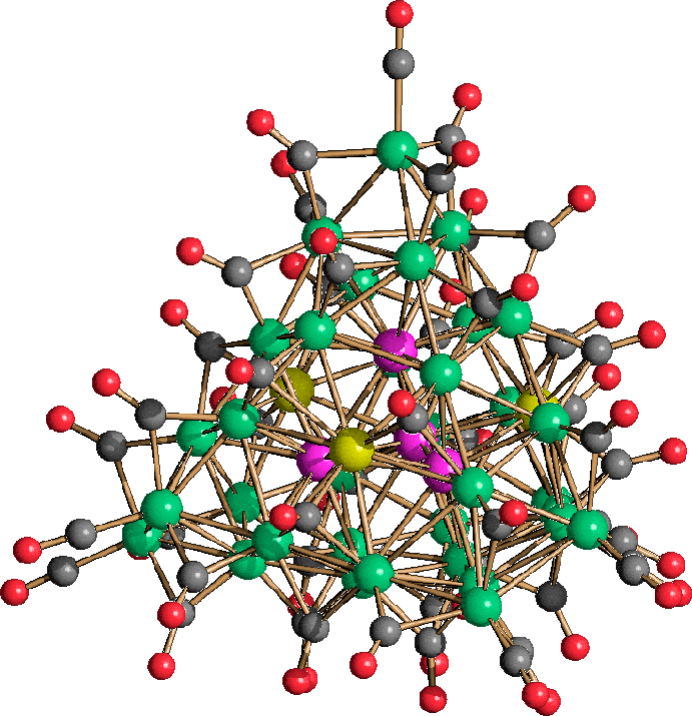
Molecular structure of **1**^**6–**^ [green for Ni, purple for Pt, yellow for Ni/Pd (≈53/47), gray for C, and red for O].

**Figure 2:**
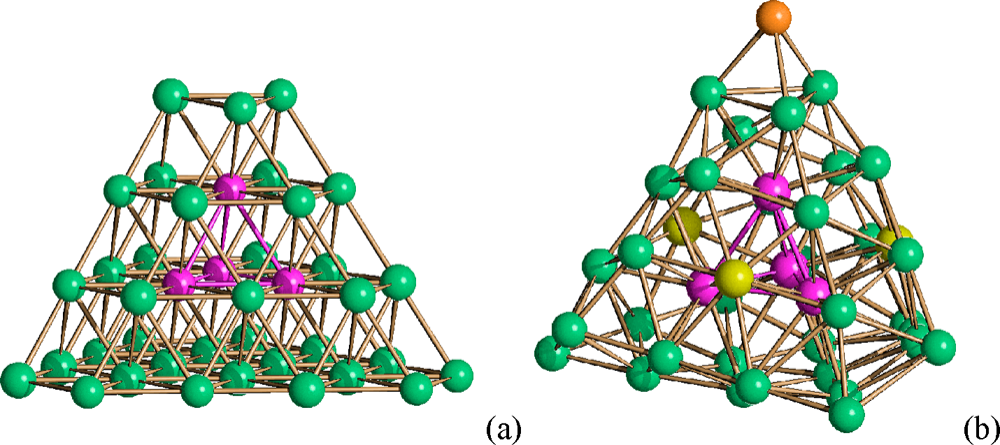
(a) Idealized *ccp* M_40_ core of **1**^**6–**^ (green for surface atoms and purple for the fully interstitial M_4_ tetrahedron) and (b) its real M_41_ metal core obtained by adding a further Ni atom (orange) to the top triangular face [yellow for three disordered Ni/Pd sites at the center of the (111) faces].

The idealized metal core of **1**^**6–**^ may be derived from a cubic close packed (*ccp*) M_40_ framework composed of four ABCA layers consisting of 3, 7, 12, and 18 atoms, respectively (Figure 1 and Table 1). This M_40_ framework encapsulates a fully interstitial Pd_4_ tetrahedron. The presence of a bulkier Pd_4_ tetrahedron within the mixed NiPd metal cage generates a large distortion of the *ccp* kernel that considerably departs from the idealized M_40_ model (Figure 2). An additional Ni atom caps the unique triangular face of the A(3) layer (Figure 2).

**Table 1:** Main Bond Distances (angstroms) of **1**^**6–**^, **3**^**5–**^, and **2**^**6–**^ Compared to Those of [Ni_22–*x*_Pd_20+*x*_(CO)_48_]^6–^, [Ni_29–*x*_Pd_6+*x*_(CO)_42_]^6–^, [Ni_29+*x*_Pd_6–*x*_(CO)_42_]^6–^, and [Ni_37_Pt_4_(CO)_46_]^6–^

	Ni–Ni	Ni–M^a^	M–M^a^
**1**^**6–**^	2.401(3)–3.006(3)	2.400(2)–2.796(2)	2.695(2)–2.722(2)
	average 2.57(2)	average 2.634(11)	average 2.713(5)
**3**^**5–**^	2.373(4)–2.808(4)	2.497(3)–2.844(3)	2.618(3)–2.844(3)
	average 2.57(3)	average 2.60(3)	average 2.696(18)
**2**^**6–**^	2.3754(17)–2.8280(17)	2.4995(16)–2.8537(13)	2.6412(13)–2.8537(13)
	average 2.598(9)	average 2.634(7)	average 2.735(4)
[Ni_22–*x*_Pd_20+*x*_(CO)_48_]^6–^b	2.4696(10)–2.95(2)	2.40(4)–2.82(3)	2.59(3)–2.9053(6)
	average 2.56(4)	average 2.67(5)	average 2.773(19)
[Ni_29–*x*_Pd_6+*x*_(CO)_42_]^6–^b	2.444(3)–2.725(3)	2.35(7)–2.68(7)	2.618(2)–2.7689(15)
	average 2.58(4)	average 2.61(9)	average 2.68(3)
[Ni_29+*x*_Pd_6–*x*_(CO)_42_]^6–^b	2.437(5)–2.732(3)	2.324(17)–2.889(17)	2.6256(19)–2.7585(13)
	average 2.58(2)	average 2.65(5)	average 2.704(3)
[Ni_37_Pt_4_(CO)_46_]^6–^c	2.421(2)–2.955(2)	2.536(2)–2.776(2)	2.709(2)–2.733(2)
	average 2.562(17)	average 2.641(8)	average 2.724(5)

a M = Pd or Pt.

b See ref (14).

c See refs (49) and (50).

A similar structure has been previously reported for the related Ni–Pt species [Ni_37_Pt_4_(CO)_46_]^6–^.^49,50^ The main difference between **1**^**6–**^ and [Ni_37_Pt_4_(CO)_46_]^6–^ is that in the latter there is a perfect segregation of Ni and Pt, all four Pt atoms being fully interstitial and all Ni atoms present on the surface of the cluster. Conversely, in the case of **1**^**6–**^, beside the fully interstitial Pd_4_ tetrahedron, there is an additional Pd atom disordered over three symmetry-related (by a 3-fold axis) positions. These three positions are located at the center of the three distorted (111) faces of the metal cage of the cluster. A free refinement of the occupancy factor of the independent site resulted in 0.526(18) Ni and 0.474(18) Pd. Because of the 3-fold symmetry, this corresponds to 1.59 Ni and 1.41 Pd atoms per cluster unit. Thus, **1**^**6–**^ displays both substitutional and compositional disorder, being actually a mixture of [Ni_36_Pd_5_(CO)_46_]^6–^ (59%) and [Ni_35_Pd_6_(CO)_46_]^6–^ (41%). It must be mentioned that the surface Pd atoms are at the center of almost flat centered hexagonal Ni_6_Pd faces, displaying high M–M and low M–CO connectivities, as previously found in other Ni–Pd carbonyl clusters (Table 2).

**Table 2:** M–M and M–CO (M = Ni or Pd) Connectivities of Ni–Pd Carbonyl Clusters Reported as Intervals

	Ni		Pd	
	Ni–M	Ni–CO	Pd–M	Pd–CO
[Ni_13_Pd_13_(CO)_34_]^4–^a	3–7	3	6–12	2
[Ni_16_Pd_16_(CO)_40_]^4–^b	5–6	3–4	7–12	0–2
[Ni_29–*x*_Pd_6+*x*_(CO)_42_]^6–^c	4–8	2–3	8–12	0–2
[Ni_29+*x*_Pd_6–*x*_(CO)_42_]^6–^c	4–8	2–3	8–12	0–3
**1**^**6–**^	3–8	2–4	8–12	0–3
[Ni_22–*x*_Pd_20+*x*_(CO)_48_]^6–^c	3–9	3–4	8–12	0–3
**3**^**5–**^	4–9	2–3	9–12	0–3
**2**^**6–**^	4–9	2–3	9–12	0–3
[Ni_36_Pd_8_(CO)_48_]^6–^d	4–9	2–3	9–12	0–2

a See ref (46).

b See ref (47).

c See ref (14).

d See ref (45).

The metal core of **1**^**6–**^ contains 150 M–M bonds and is completed by 46 CO ligands (seven terminal, 26 edge-bridging, and 13 face-bridging). The cluster displays 508 cluster valence electrons [CVEs; 36 × 10 (Ni) + 5 × 10 (Pd) + 46 × 2 (CO) + 6 (charge)] that correspond to 6*n* + 8 (*n* is the number of metal atoms) cluster valence orbitals (CVOs) assuming that each orbital is filled with two electrons.^2^ This electron count is in keeping with other Ni–Pd carbonyl clusters. Thus, [Ni_13_Pd_13_(CO)_34_]^4–^,^46^ [Ni_16_Pd_16_(CO)_40_]^4–^,^47^ [Ni_29–*x*_Pd_6+*x*_(CO)_42_]^6–^ (*x* = 0.09), and [Ni_29+*x*_Pd_6–*x*_(CO)_42_]^6–^ (*x* = 0.27)^14^ display 6*n* + 10 CVOs; [Ni_26_Pd_20_(CO)_54_]^6–^ displays 6*n* + 11 CVOs,^47^ [Ni_22–*x*_Pd_20+*x*_(CO)_48_]^6–^ (*x* = 0.63) 6*n* + 9 CVOs,^14^ and [Ni_36_Pd_8_(CO)_48_]^6–^ 6*n* + 7 CVOs.^45^ It must be mentioned that this is just an empirical rule for electron bookkeeping in HNMCCs, where the CVE number merely results from the sum of the valence electrons of each metal (including d electrons for transition metals), the electrons arising from the ligands (two for CO), and the total charge of the cluster.^2^ The number of filled CVOs is obtained by dividing the CVE number by 2, thus assuming two paired electrons per orbital (diamagnetic cluster). The CVE number can be determined exactly, once the cluster formula is known. In contrast, the real CVO number may be greater than the calculated one, in the case of paramagnetic clusters. At the moment, there is no general rule for predicting *a priori* the CVE and CVO numbers for HNMCCs.^1−5^ As mentioned above, it has been empirically observed that for all of the HNMCCs characterized to date, the calculated CVE number corresponds to 6*n* + *x* CVOs (*n* is the number of metal atoms and *x* is an empirical number comprised in the range 2 to 24) when *x* can assume all values between 2 and 24. Even if the empirical parameter *x* is within a wide range, it displays similar values for related clusters. Thus, for Ni–Pd carbonyl clusters, *x* is between 7 and 11,^14,45−47^ whereas it can reach higher values in the case of HNMCCs containing interstitial main group atoms such as phosphides and carbides.^51,52^

**Figure 3:**
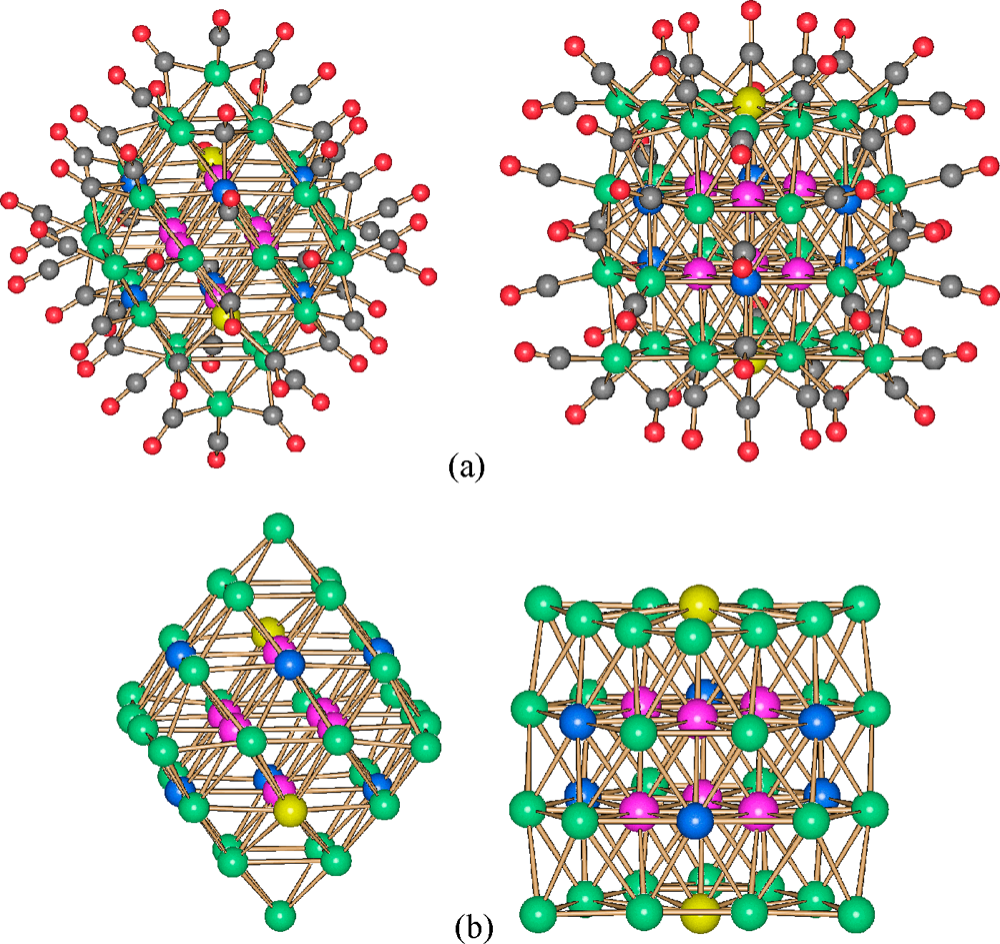
(a) Molecular structure of **2**^**6–**^ and (b) its M_44_ metal core. Two views are shown (green for Ni, purple for Pt, yellow for Ni/Pd (≈33/67), blue for Ni/Pd (≈91/9), gray for C, and red for O).

The presence of substitutional and compositional disorder in Ni–Pd carbonyl clusters is further exemplified by the structures of **2**^**6–**^ and **3**^**5–**^ (Figure 3, Table 1, and Figures S1 and S2). The related [Ni_36_Pd_8_(CO)_48_]^6–^ anion has been previously reported.^45^ Its metal framework consists of a Ni_36_Pd_8_ ν_3_ octahedron (i.e., an octahedron with a frequency of 3) encapsulating a Pd_6_ octahedron. The two additional Pd atoms on the surface are disordered, showing a preference for the central position of the eight (111) faces. The **2**^**6–**^ and **3**^**5–**^ anions reported herein display rather similar structures, and in addition to Ni/Pd substitutional disorder, they show compositional disorder. Thus, **2**^**6–**^ is actually a mixture of [Ni_37_Pd_7_(CO)_48_]^6–^ (31%) and [Ni_36_Pd_8_(CO)_48_]^6–^ (69%), whereas **3**^**5–**^ consists of [HNi_37_Pd_7_(CO)_48_]^5–^ (47%) and [HNi_36_Pd_8_(CO)_48_]^5–^ (53%). The overall Ni/Pd distribution in both clusters is very similar to that previously observed for [Ni_36_Pd_8_(CO)_48_]^6–^. Thus, the six fully interstitial positions are occupied by Pd atoms, and the additional Pd atoms are disordered at the center of the eight (111) hexagonal faces. Some minor differences in the occupancy factors of these sites are observed for the different species. In addition, in the case of **3**^**5–**^, two independent molecules are present within the unit cell, displaying some significant differences in their compositions. Thus, the first independent molecule consists of [HNi_37_Pd_7_(CO)_48_]^5–^ (26%) and [HNi_36_Pd_8_(CO)_48_]^5–^ (74%), whereas the second site contains [HNi_37_Pd_7_(CO)_48_]^5–^ (68%) and [HNi_36_Pd_8_(CO)_48_]^5–^ (32%).

The metal cores of **2**^**6–**^ and **3**^**5–**^ contain 168 M–M bonds and are completed by 48 CO ligands (18 terminal, 12 edge-bridging, and 18 face-bridging). They both display 542 CVEs [37 × 10 (Ni) + 7 × 10 (Pd) + 48 × 2 (CO) + 6 (charge) for **2**^**6–**^; 37 × 10 (Ni) + 7 × 10 (Pd) + 48 × 2 (CO) + 1 × 1 (H) + 5 (charge) for **3**^**5–**^] and 271 CVOs as previously found for [Ni_36_Pd_8_(CO)_48_]^6–^ [36 × 10 (Ni) + 8 × 10 (Pd) + 48 × 2 (CO) + 6 (charge)].

### 2.2 Electrochemistry and Infrared Spectroelectrochemistry

#### 2.2.1 [Ni_36–*x*_Pd_5+*x*_(CO)_46_]^6–^ (*x* = 0.41) (**1**^**6–**^)

**Figure 4:**
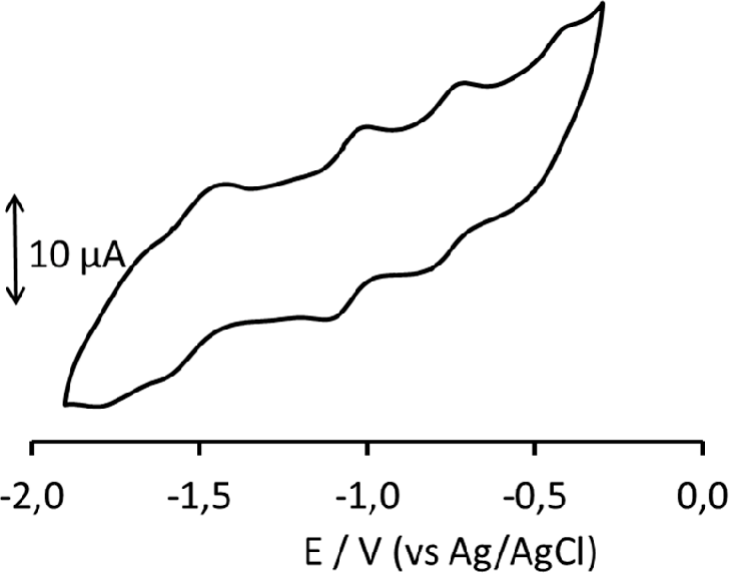
CV profile recorded at the Pt electrode in a CH_3_CN solution of **1**^**6–**^. [NBu_4_][PF_6_] (0.1 mol dm^–3^) as the supporting electrolyte. Scan rate of 0.1 V s^–1^.

The CV profile of **1**^**6–**^ in a CH_3_CN/[NBu_4_][PF_6_] solution, although not so well resolved as in the case of mononuclear complexes, is not so poorly defined as often found with other multivalent HNMCCs (Figure 4).^14−18^ The same CV profile was obtained on Pt or GC WEs. Two resolved redox processes, which appear to be chemically reversible, are present at −0.76 and −1.05 V (vs Ag/AgCl, KCl saturated). Two other partially overlapping processes are detected at more cathodic potentials (−1.54 and −1.79 V), and another not very resolved peak is observed at −0.46 V (Figure 4 and Table 3). On the basis of the IR SEC analysis, it has been possible to establish that **1**^**6–**^ undergoes three chemically reversible reductions on the time scale of the IR SEC experiment (Figures S3–S5), while the process at −1.79 V is accompanied by an extensive decomposition of the electrogenerated species, as indicated by the appearance and fast increase in the intensity of the band at 2127 cm^–1^ due to dissolved CO.

**Table 3:** Peak Potential Values (volts, vs Ag/AgCl, KCl saturated) for the Redox Changes Exhibited by Clusters **1**^**6–**^, **3**^**5–**^, and **2**^**6–**^ in a CH_3_CN/[NBu_4_][PF_6_] Solution^a^

	redox steps						
complex	4– to 5–b	5– to 6–b	6– to 7–b	7– to 8–b	8– to 9–b	9– to 10–b	10– to 11–b
**1**^**6–**^		–0.46^c^	–0.76^c^	–1.05^c^	–1.54^c^	–1.79	
**3**^**5–**^	–0.19^c^	–0.52^c^	–0.89^c^	–1.22^c^	–1.56	–1.91	
**2**^**6–**^		–0.57^c^	–0.75^c^	–1.08^c^	–1.26^c^	–1.61	–1.87

a Data from differential pulse voltammetry at 0.02 V s^–1^.

b One-electron process, as inferred from IR SEC.

c Chemically reversible processes on the time scale of IR SEC.

A shift at higher frequencies of the two IR bands of **1**^**6–**^ [from 1993 and 1856 cm^–1^ to 2010 and 1868 cm^–1^, respectively (Figure S6)] indicates that the peak at −0.46 V corresponds to an oxidation. However, before the complete oxidation of **1**^**6–**^, a weak shoulder at 2043 cm^–1^ due to Ni(CO)_4_ indicates a relatively slow fragmentation of the metallic core of the electrogenerated [Ni_36–*x*_Pd_5+*x*_(CO)_46_]^5–^ (**1**^**5–**^). The starting spectrum of **1**^**6–**^ was almost completely restored in the reverse reduction back scan.

The IR SEC study allowed us to identify five redox couples between −0.24 and −1.80 V (vs the Ag pseudoreference electrode). One oxidation step produced a cluster of limited stability; three reductions, chemically reversible in the time scale of the IR SEC experiment, demonstrated that the core of the cluster is stable with up to three additional electrons, while the addition of a fourth electron caused a relatively fast CO evolution.

**Figure 5:**
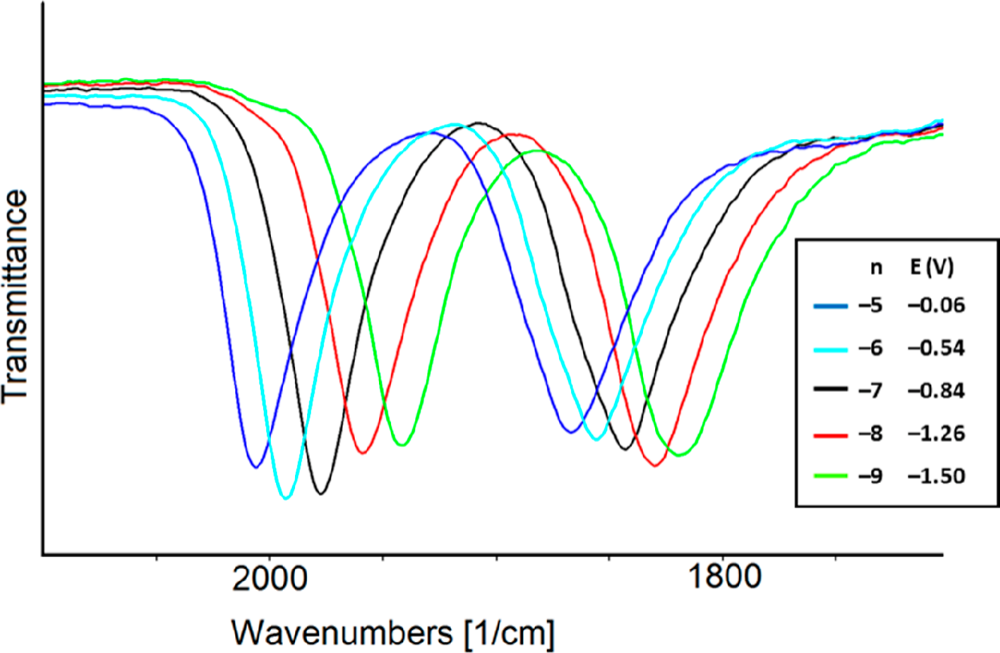
Selected IR spectra of **1**^***n*–**^ as a function of cluster charge *n* and potential *E* (volts, vs the Ag pseudoreference electrode) in CH_3_CN containing 0.1 mol dm^–3^ [NBu_4_][PF_6_]. The absorptions of the solvent and supporting electrolyte have been subtracted.

The IR spectra of the five oxidation states of [Ni_36–*x*_Pd_5+*x*_(CO)_46_]^*n*−^ (*n* = 5–9) (**1**^***n*–**^) were selected (Figure 5), and the charge of each species was assigned according to one-electron transfer for each redox exchange, as suggested by a near-uniform shift of 15–19 cm^–1^ of the stretching frequencies of ν^t^_CO_ for all processes.^14,20−23^ The stretching frequencies of the terminal (ν^t^_CO_) and bridging (ν^b^_CO_) carbonyl groups for each species are listed in Table 4. A propensity for an increase in the intensity of the ν^b^_CO_ bands compared to the ν^t^_CO_ bands was observed during the consecutive reduction steps. This pointed to some structural changes regarding the stereochemistry of CO ligands for species with increased negative charge and may foresee a slowdown of the electron transfers that is a decrease in the electrochemical reversibility for the related redox steps.

**Table 4:** IR Frequencies (cm^–1^) of the Terminal (ν^t^_CO_) and Bridging (ν^b^_CO_) Carbonyl Groups for **1**^***n*–**^ in CH_3_CN as a Function of Cluster Charge *n*

cluster charge *n*	ν^t^_CO_	ν^b^_CO_
–5	2010	1868
–6	1993	1856
–7	1978	1843
–8	1959	1830
–9	1942	1819

#### 2.2.2 [HNi_37–*x*_Pd_7+*x*_(CO)_48_]^5–^ (*x* = 0.53) (**3**^**5–**^)

**Figure 6:**
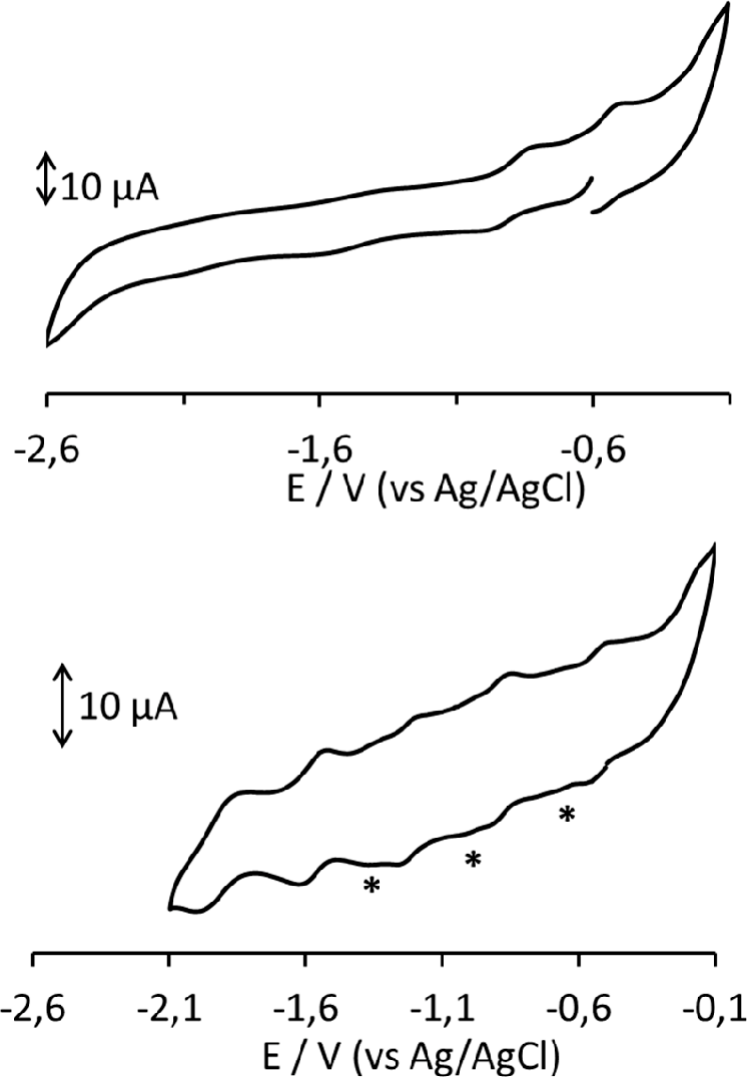
CV profiles recorded at Pt (top) and GC (bottom) electrodes in a CH_3_CN solution of **3**^**5–**^. [NBu_4_][PF_6_] (0.1 mol dm^–3^) as the supporting electrolyte. Scan rate of 0.1 V s^–1^. Starred peaks can be attributed to unknown impurities.

The CV profiles of **3**^**5–**^ in a CH_3_CN/[NBu_4_][PF_6_] solution at different Pt electrodes were always less resolved than those at the GC WE. Five processes, all possessing features of chemical reversibility on the CV time scale, are observed between −0.3 and −2.1 V (vs Ag/AgCl, KCl saturated) at the GC WE (Figure 6 and Table 3), while only two processes can be detected at a Pt WE in the same potential range. Between −0.3 and −0.1 V, the profiles on both electrodes showed a current increase without a well-resolved peak, presumably indicating the occurrence of other redox changes. The starred peaks in Figure 6 and in the differential pulse voltammogram shown in Figure S7 were due to unknown impurities.

When the redox chemistry of **3**^**5–**^ was studied by IR SEC in an OTTLE cell, we found that the potential can be cycled between 0.0 and −1.6 V (vs the Ag pseudoreference electrode) without any decomposition of the electrogenerated species. Indeed, the IR spectrum of the starting cluster was restored when the WE potential was returned to the initial value. From the analysis of the complete sequence of the recorded IR spectra, it has been possible to identify five redox states of **3**^**5–**^. In particular, during the slow sweep of the WE potential from −0.21 to −1.60 V, the ν_CO_ bands of **3**^**5–**^ (2002 and 1860 cm^–1^) were shifted to lower wavenumbers and three reduction processes, completely reversible on the time scale of IR SEC, were observed (Figures S8–S10). With a further decrease in the applied potential from −1.6 to −2.0 V, the shift of the ν_CO_ bands at lower wavenumbers was accompanied by a clear variation of the relative intensity of the terminal and bridging ν_CO_ bands in favor of the bridging ones (Figure S11). This is in agreement with the fact that with an increase in the negative charge of a carbonyl cluster, bridging CO ligands are more favored than terminal ones.^24,25^ A similar change in the stereochemistry of the CO ligands was previously observed for other clusters. This change was related to the electrochemical quasi-reversibility of the redox processes, which retained the chemical reversibility because a spectrum that could be superimposed with that of the starting cluster was obtained when the WE potential was returned to the initial value.^14−18,20−23^ In the case of **3**^**5–**^, instead, during the back oxidation scan up to −0.21 V, the shift of the ν_CO_ bands at the initial values was observed, but not equally restored was their intensity ratio.

With a further decrease in the WE potential from −2.0 to −2.2 V, CO release in solution and other modifications of the IR spectra suggested incipient decomposition of the multireduced cluster (Figure S12).

When the WE potential was increased from −0.21 to 0.1 V (vs the Ag pseudoreference electrode), an oxidation of the cluster was indicated by the upshift of the ν_CO_ bands at 2013 and 1870 cm^–1^. However, the formation of Ni(CO)_4_ starting before the complete oxidation of the initial cluster indicated the limited stability of the oxidized species (Figure S13), and the spectrum of **3**^**5–**^ was not completely restored in the reverse reduction back scan (Figure S14).

**Figure 7:**
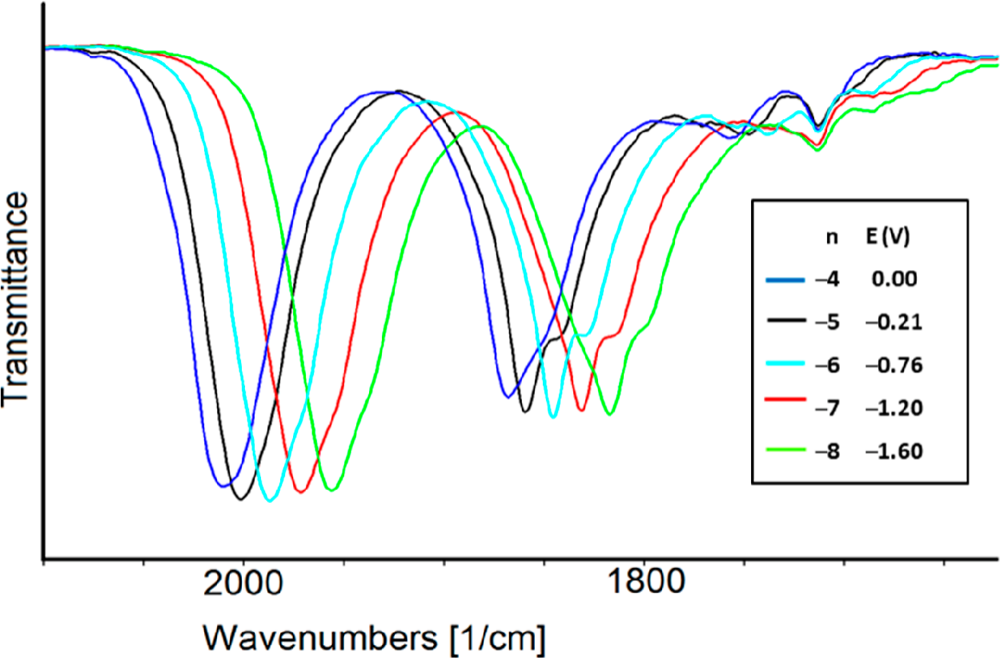
Selected infrared spectra of **3**^***n*–**^ as a function of cluster charge *n* and potential *E* (volts, vs the Ag pseudoreference electrode) in CH_3_CN containing 0.1 mol dm^–3^ [NBu_4_][PF_6_]. The absorptions of the solvent and supporting electrolyte have been subtracted.

The vibrational spectra in the ν_CO_ region of the five reversible redox states of cluster [HNi_37–*x*_Pd_7+*x*_(CO)_48_]^*n*−^ (*n* = 4–8) (**3**^***n*–**^) are shown in Figure 7, and the ν^t^_CO_ and ν^b^_CO_ stretching frequencies for each species are listed in Table 5. On the basis of the shift of the ν^t^_CO_ bands (∼15 cm^–1^), all of the processes were considered monoelectronic and the charge of each species was consequently assigned.

**Table 5:** Infrared Stretching Frequencies (cm^–1^) of the Terminal (ν^t^_CO_) and Bridging (ν^b^_CO_) Carbonyl Groups for **3**^***n*–**^ in CH_3_CN as a Function of Cluster Charge *n*

cluster charge *n*	ν^t^_CO_	ν^b^_CO_
–4	2013	1870
–5	2002	1860
–6	1987	1846
–7	1972	1832
–8	1957	1818

#### 2.2.3 [Ni_37–*x*_Pd_7+*x*_(CO)_48_]^6–^ (*x* = 0.69) (**2**^**6–**^)

**Figure 8:**
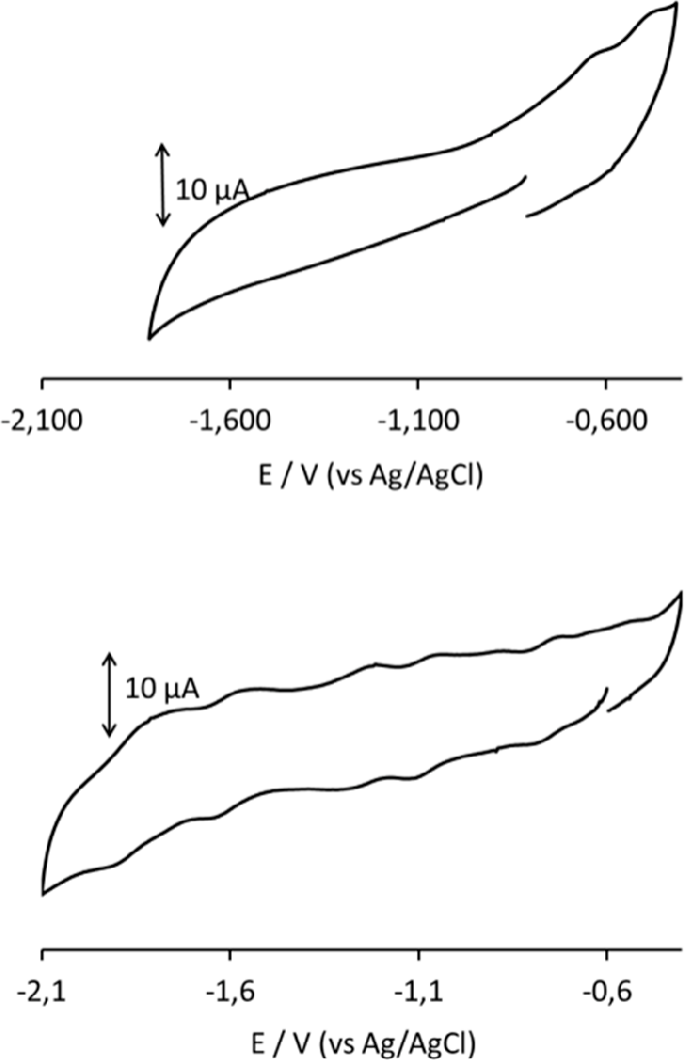
CV profile recorded at Pt (top) and GC (bottom) electrodes in a CH_3_CN solution of **2**^**6–**^. [NBu_4_][PF_6_] (0.1 mol dm^–3^) as the supporting electrolyte. Scan rate of 0.2 V s^–1^.

The cyclic voltammograms of **2**^**6–**^ at GC and Pt electrodes are shown in Figure 8. Several redox processes having features of chemical reversibility on the CV time scale can be detected on only the GC electrode. Also in this case, the presence of impurities made the weak peaks less defined. However, by a comparison with the CV profile of protonated cluster **3**^**5–**^ (Figure S15), it seemed that the two species were not the same cluster with different oxidation states, thus supporting the presence of a hydride atom in **3**^**5–**^. Moreover, we excluded the possibility that the impurities present in both samples could be attributed to protonation–deprotonation equilibria often observed for similar Ni–Pt or Ni–Pd clusters.^45,50,53^

**Figure 9:**
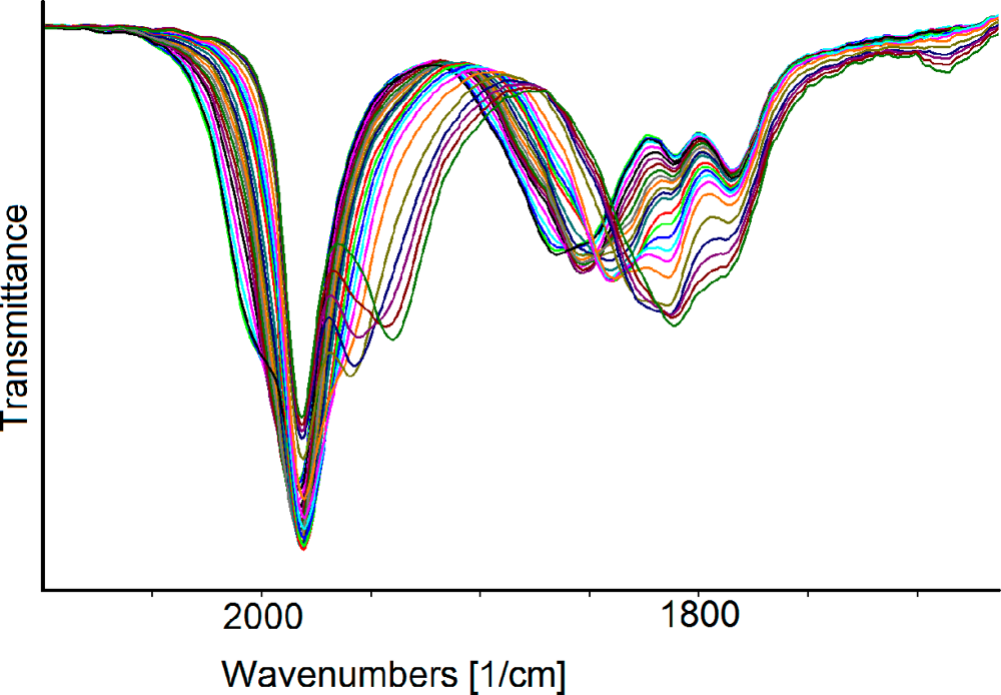
IR spectral changes of a CH_3_CN solution of **2**^**6–**^ recorded in an OTTLE cell during the progressive sweep of the potential from −0.3 to −1.8 V vs the Ag pseudoreference electrode (scan rate of 1 mV s^–1^). [NBu_4_][PF_6_] (0.1 mol dm^–3^) as the supporting electrolyte. The absorptions of the solvent and the supporting electrolyte have been subtracted.

Small amounts of [Ni_6_(CO)_12_]^2–^ were found in the sample of **2**^**6–**^ used for the CV and IR SEC investigations. Such an impurity is not observed in the CV of Figure 8 between −2.1 and −0.4 V, because its reversible reduction occurs at −2.31 V and an irreversible oxidation at −0.31 V. Instead, the presence of [Ni_6_(CO)_12_]^2–^ is evident in the IR SEC experiments. Figure 9 reports the IR spectra recorded in an OTTLE cell containing a CH_3_CN solution of **2**^**6–**^ when the WE potential is swept between −0.3 and −1.8 V (vs the Ag pseudoreference electrode). While a downshift of ν_CO_ bands with a decrease in the potentials is clearly evident in the bridging CO region, the overlap of ν^t^_CO_ bands belonging to **2**^**6–**^ (1990 and 1953 cm^–1^) and [Ni_6_(CO)_12_]^2–^ (1982, 1810, and 1784 cm^–1^) made it difficult to detect in this IR region the real shift induced by the redox processes occurring from −0.3 to −1.8 V (vs the Ag pseudoreference electrode). On the basis of the consecutive shift of the ν^b^_CO_ bands of **2**^**6–**^ from 1865 to 1853, 1840, 1826, and 1812 cm^–1^, the IR spectra shown in sequence in Figure 9 were separated into four groups, each of them attributable to a reversible redox process. To eliminate the [Ni_6_(CO)_12_]^2–^ interfering IR bands, which remained unchanged in the applied potential window, we calculated the differential absorbance spectra using as a reference spectrum the first one in each group. The four IR spectral changes observed in the potential range from −0.3 to −1.8 V are shown in Figures S16–S19. When the WE potential was increased from −0.6 to −0.3 V, an upshift of both ν_CO_ bands of **2**^**6–**^ pointed to an oxidation process, while with a decrease in the potential from −0.6 to −1.8 V, three consecutive downshifts of both IR absorptions of **2**^**6–**^ were attributed to three reduction processes. All of the redox steps were completely chemically reversible, and the potential could be cycled between −0.3 and −1.8 V without decomposition of the electrogenerated species. Indeed, the IR spectrum of the starting **2**^**6–**^ was obtained when the WE potential was returned to the initial value (Figure S20). Instead, with an increase in the potential above −0.3 V or a decrease to values more negative than −1.8 V, relatively fast decomposition reactions were observed.

Even if the results of the IR SEC study are not as well resolved as those for **1**^**6–**^ and **3**^**5–**^, we can conclude that **2**^**6–**^ is also stable, on the time scale of the IR SEC experiment, with a variable number of electrons.

### 2.3 Electrochemical Impedance Spectroscopy (EIS)

**Figure 10:**
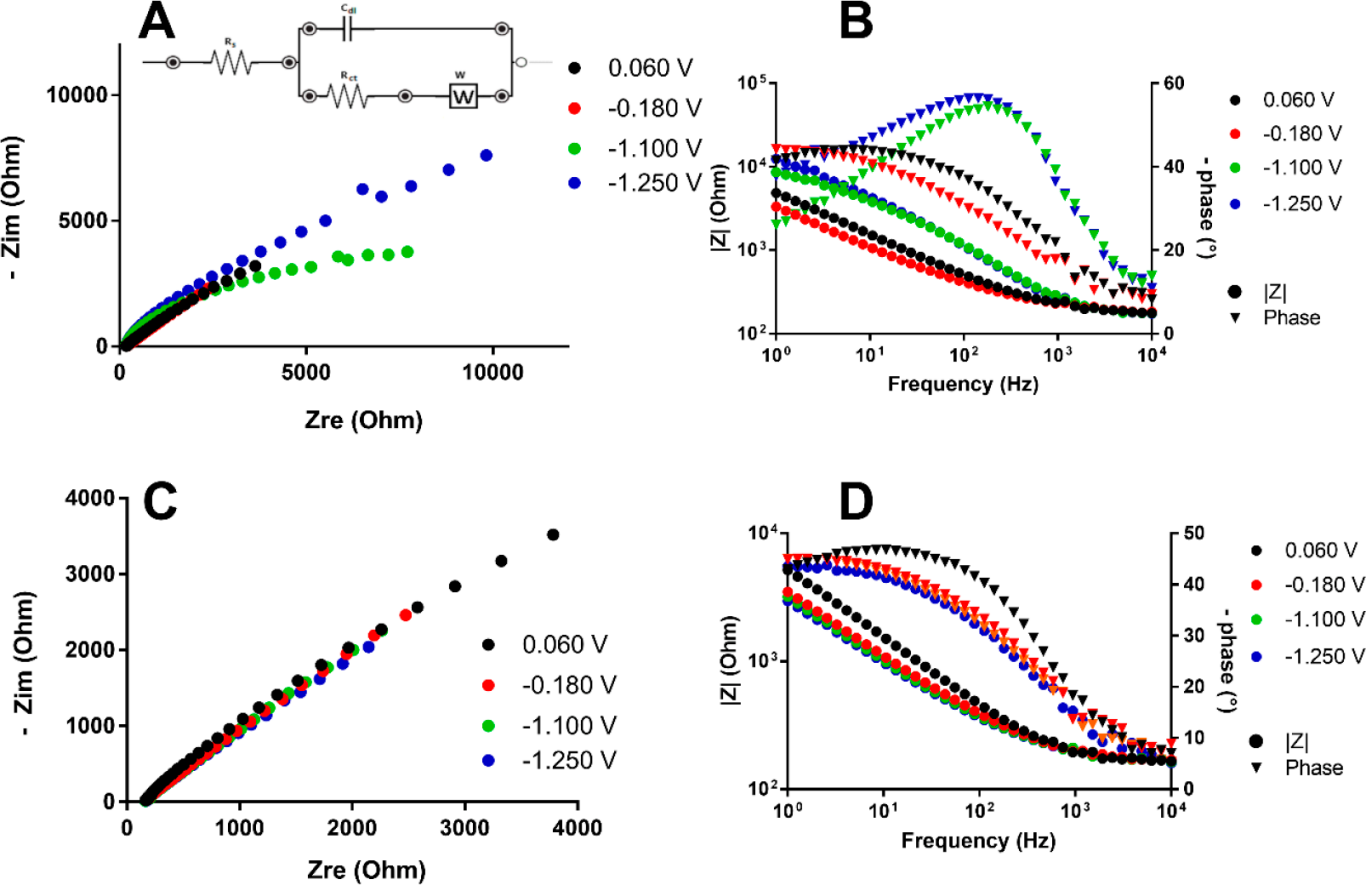
(A) Nyquist plot for [Pt_19_(CO)_22_]^4–^ using a Pt electrode. (B) Bode plot using a Pt electrode. (C) Nyquist plot for [Pt_19_(CO)_22_]^4–^ using a GC electrode. (D) Bode plot using a GC electrode. The working DC potentials for each curve were 0.060 V (black circles), −0.180 V (red circles), −1.100 V (green circles), and −1.250 V (blue circles).

To further investigate the electrochemical behavior of **1**^**6–**^, **2**^**6–**^, and **3**^**5–**^, EIS measurements were performed. In EIS experiments, a small ac voltage with a variable frequency is superimposed on a dc voltage applied to the cell, and the corresponding impedance is recorded.^54^ With a change in the frequency, phenomena taking place at the electrode–solution interface can be discriminated and separated. To better describe such phenomena, the electrochemical cell can be schematized by the use of an equivalent circuit describing the frequency-dependent components of the cell. The most widely used equivalent circuit to describe a cell containing an electroactive species is the Randles circuit, shown in Figure 10A. In this diagram, *R*_s_ represents the resistance of the solution, *C*_dl_ is the double-layer capacitor generated by charges at the electrode–solution interface, *R*_ct_ is the charge transfer resistance during a redox process, and *W*, called the Warburg impedance, represents the impedance related to the diffusion process. A nonlinear fit can be performed on the experimental data to obtain the corresponding value of the equivalent circuit elements. Because to the best of our knowledge, this is the first time that EIS is used to analyze redox processes of metal carbonyl clusters, we decided at first to study a metallic cluster whose redox chemistry is available in the literature, that is [Pt_19_(CO)_22_]^4–^.^20^ The behavior of *R*_ct_ at the *E*° potential of electron transfer is directly related to the kinetics of the electrochemical reaction (eq 1) and was used to devise the overall kinetic behavior of the cluster toward electron transfer:
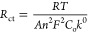
1where *R* is the gas constant, *T* is the temperature, *n* is the number of transferred electrons, *F* is the Faraday constant, *k*^0^ is the heterogeneous rate constant, *C*_o_ is the concentration of the species under investigation, and *A* is the area of the electrode. Furthermore, the experiments were performed using both GC and Pt WEs to investigate the different kinetics on each electrode type. For this second analysis, preliminary CV using a known concentration of ferrocene (1.15 × 10^–3^ M) was performed to verify the similar dimensions of the electrode electroactive area (see Experimental Section) and, thus, the comparability of the *R*_ct_ value.

The CV responses between 0.2 and −1.6 V of a CH_3_CN/[NBu_4_][PF_6_] solution of [Pt_19_(CO)_22_]^4–^ recorded at a GC and at a Pt WE are shown in Figure S21 and were used to determine the *E*° values for EIS measurements. As shown in Figure 10, the Nyquist (negative imaginary part of the impedance vs the real part) and Bode (log|*Z*| and phase shift angle Φ vs the logarithm of the frequency) plots of the impedance spectra recorded at the *E*° values of successive redox peaks display a negligible difference in the profiles when Pt and GC electrodes are compared for the peaks at 0.06 and −0.18 V. Apparently, the corresponding calculated *R*_ct_ values, obtained by fitting the experimental data with the Randles circuit using the integrated tool of PStrace version 5.8, are similar (∼1 kΩ) and indicate relatively fast electron transfers for both electrodes. On the contrary, the peaks at −1.10 and −1.25 V show a higher impedance value for the Pt electrodes (*R*_ct_ ∼ 5 kΩ), suggesting slower kinetics for such electrodes, as it was supposed from lower and broader current peaks in CV (Figure S21).

**Figure 11:**
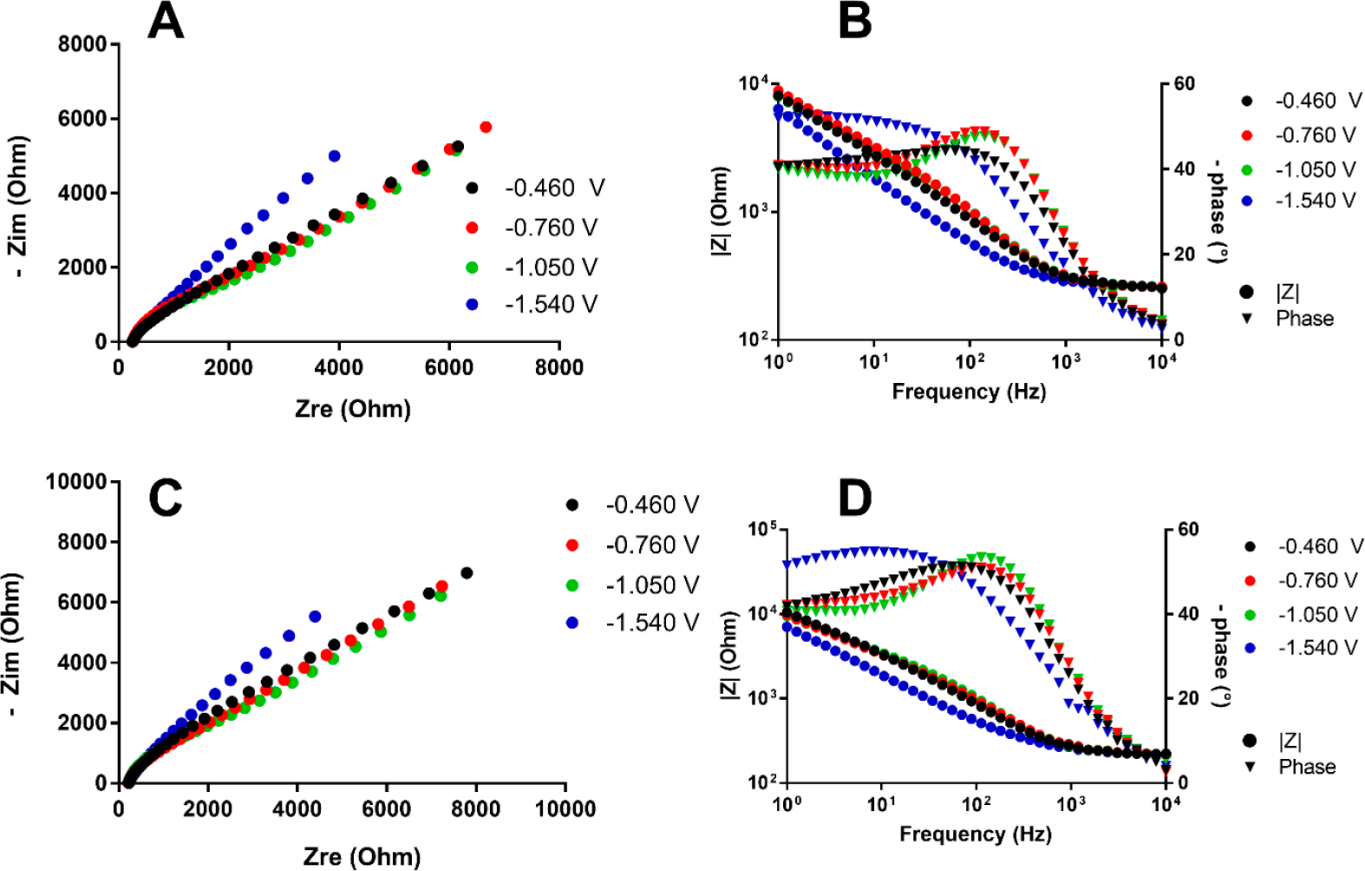
(A) Nyquist plot for **1**^**6–**^ using a Pt electrode. (B) Bode plot using a Pt electrode. (C) Nyquist plot for **1**^**6–**^ using a GC electrode. (D) Bode plot using a GC electrode. The working potentials for each curve were −0.460 V (black circles), −0.760 V (red circles), −1.050 V (green circles), and −1.540 V (blue circles).

**Figure 12:**
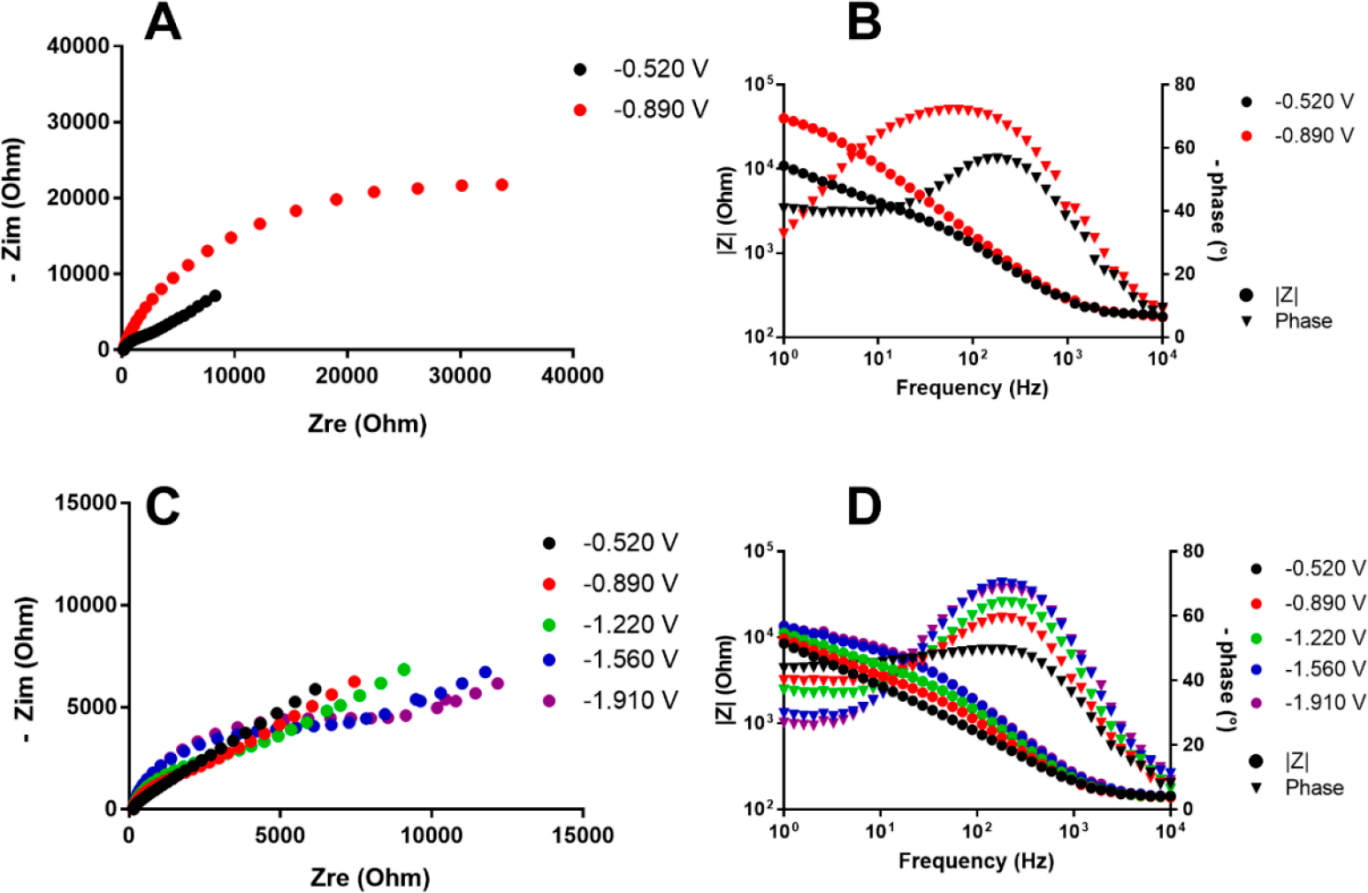
(A) Nyquist plot for **3**^**5–**^ using a Pt electrode. (B) Bode plot using a Pt electrode. (C) Nyquist plot for **3**^**5–**^ using a GC electrode. (D) Bode plot using a GC electrode. The working potentials for each curve were −0.520 V (black circles), −0.890 V (red circles), −1.220 V (green circles), −1.560 V (blue circles), and −1.910 V (purple circles).

**Figure 13:**
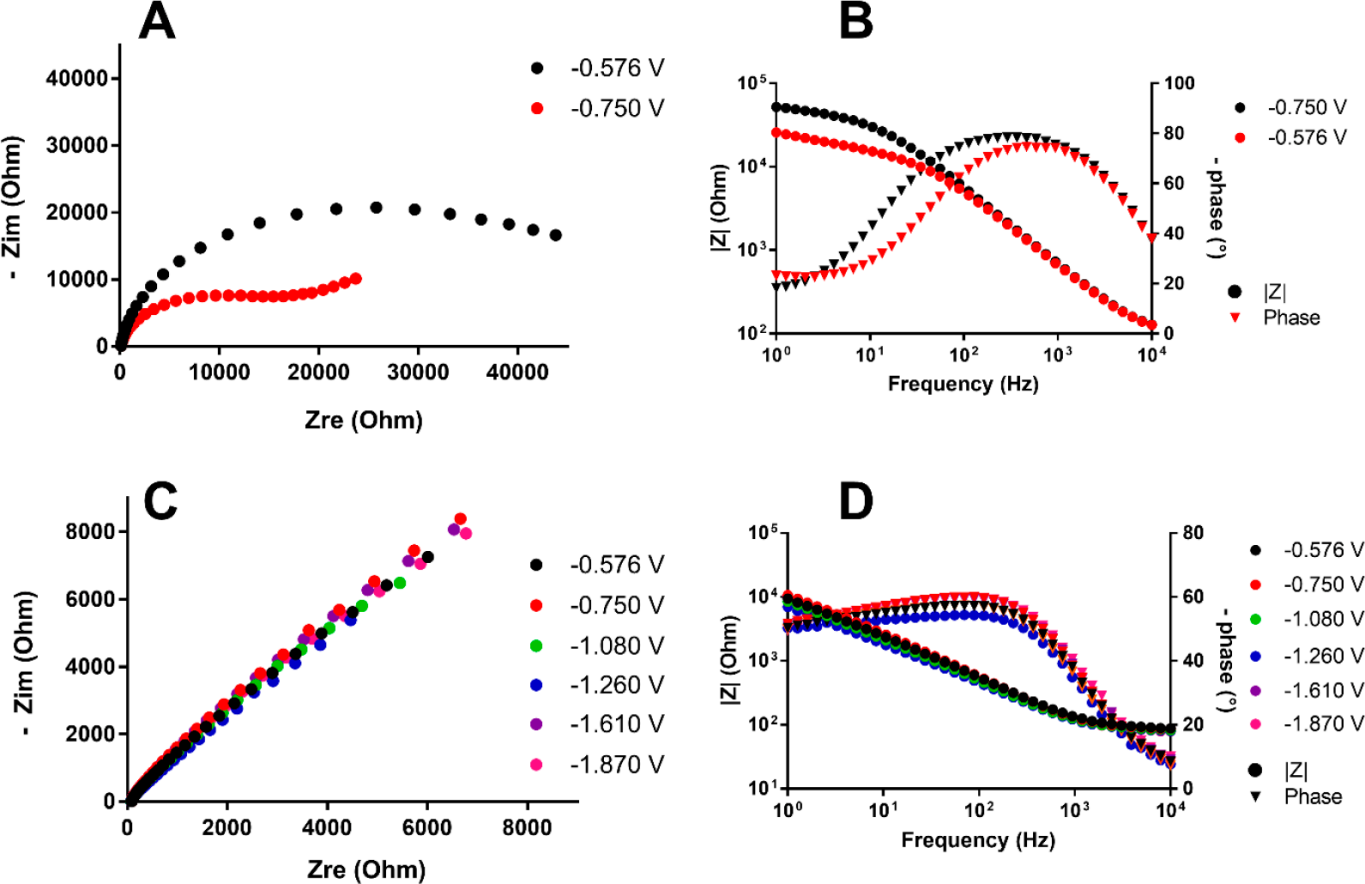
(A) Nyquist plot for **2**^**6–**^ using a Pt electrode. (B) Bode plot using a Pt electrode. (C) Nyquist plot for **2**^**6–**^ using a GC electrode. (D) Bode plot using a GC electrode. The working potentials for each curve were −0.576 V (black circles), −0.750 V (red circles), −1.080 V (green circles), −1.260 V (blue circles), −1.610 V (purple circles), and −1.870 V (pink circles).

A similar approach was used to study **1**^**6–**^, **3**^**5–**^, and **2**^**6–**^. The EIS behaviors of **1**^**6–**^, **3**^**5–**^, and **2**^**6–**^ at Pt and GC electrodes are compared in Figures 11–13.

As shown by the plots of Figure 11, **1**^**6–**^ exhibited the same behavior on both Pt and GC electrodes for all of the examined electron transfers, featuring relatively fast kinetics, with *R*_ct_ values without significant differences between voltage peaks.

The EIS measurements for **3**^**5–**^ at the potentials of the five consecutive reduction steps on different WE materials afforded results similar to those of [Pt_19_(CO)_22_]^4–^. In particular, for **3**^**5–**^ the EIS spectra of peaks at the more negative potentials could not be recorded with a Pt WE, because in the CV analysis very low currents with Pt WE made the peaks hardly observable, while high *R*_ct_ values were obtained for the peaks at −0.520 and −0.890 V (9 and 41 kΩ, respectively), confirming slow kinetics toward that electrode material. We found that subsequent reductions lead to slower kinetic behavior for **3**^**5–**^ also on a GC WE. This can be explained by supposing important structural modification of the cluster as the number of added electrons increases. An increase in the metal–metal bond distances as the negative charge of the cluster increases is expected for multivalent carbonyl compounds.^55^ Indeed, density functional theory-optimized geometries of the multivalent family of clusters [Rh_12_E(CO)_27_]^*n–*^ (E = Ge, Sn, Sb, or Bi) indicated elongation of the Rh–Rh and Rh–E bond distances for multireduced species.^56^

Similar results were also obtained for **2**^**6–**^ when comparing Pt and GC electrodes. GC EIS spectra showed fast and comparable kinetics for all of the peaks investigated. Quite the opposite results were obtained for Pt electrodes where high *R*_ct_ values (15 and 42 kΩ) were calculated from EIS measurements at the potentials of the only two peaks visible from CV measurements (−0.57 and −0.75 V, respectively).

## 3 Conclusions

Three new atomically precise Ni–Pd alloy carbonyl nanoclusters have been synthesized, and their total (molecular) structures determined by SC-XRD. The new Ni–Pd HNMCCs **1**^**6–**^, **2**^**6–**^, and **3**^**5–**^ as well as the previously reported [Ni_36_Pd_8_(CO)_48_]^6–^^45^ are structurally related to Ni–Pt clusters [Ni_37_Pt_4_(CO)_46_]^6–^, [Ni_38_Pt_6_(CO)_48_]^6–^, and [Ni_35_Pt_9_(CO)_48_]^6–^.^49,50,53^ This is not so common, because more often Ni–Pd and Ni–Pt HNMCCs display rather different structures. The comparison of these few examples of structurally related Ni–Pd and Ni–Pt clusters is quite interesting. The overall structures of their metal cores and the stereochemistry of the CO ligands are almost identical. With regard to the Ni–Pd and Ni–Pt distributions, both Pd and Pt tend to completely occupy the fully interstitial positions, even if for different reasons. Indeed, in the case of Ni–Pd carbonyl clusters, Pd tends to minimize Pd–CO contacts, because Pd–CO bonds are considerably weaker than Ni–CO ones.^14^ Conversely, in the case of Ni–Pt clusters, Pt prefers interstitial positions to maximize Pt–Pt and Pt–Ni bonds, because Pt forms very strong metal–metal bonds. Some Ni/Pd disorder is present on the surface of Ni–Pd clusters, usually concerning positions with low M–CO connectivity. This surface disorder is more limited in Ni–Pt clusters. These results indicate that the total structures of molecular alloy M–M′ nanoclusters comprise a subtle balance of the different M–ligand, M′–ligand, M–M, M–M′, and M′–M′ bonding energies.

The three HNMCCs reported herein (**1**^**6–**^, **2**^**6–**^, and **3**^**5–**^), as well the Ni–Pd alloy nanoclusters described in our previous paper,^14^ display several chemically and electrochemically reversible one-electron redox processes, as clearly demonstrated by joint CV and IR SEC experiments. In this sense, they are multivalent and behave as electron sinks and molecular nanocapacitors. Several additional pieces of information can be obtained from detailed IR SEC analyses: (a) the appearance of chemically reversible redox processes even when the peaks in their CV profiles are very weak, (b) the number of exchanged electrons, based on the ν_CO_ shift, (c) stereochemical rearrangements of the CO ligands accompanying the reversible addition and/or removal of electrons (by comparing the relative intensity of ν^t^_CO_ vs that of ν^b^_CO_), and (d) the occurrence of irreversible processes and decomposition reactions. Point (a) has been somehow debated, because it was not very clear why in some cases very low currents were detected in CV experiments leading to almost featureless *i*/*V* profiles, whereas for the same species very clear ν_CO_ shifts were present in the IR SEC experiments suggesting well-defined reversible redox processes. To shed light on this point, we performed CV experiments with different WE materials and, for the first time, EIS analyses of **1**^**6–**^, **2**^**6–**^, and **3**^**5–**^, as well as redox active [Pt_19_(CO)_22_]^4–^, whose electrochemistry was reported in detail previously.^20^ All of these experiments demonstrated that the kinetics of the electron exchange at the electrode surface interfaces is a critical point. For some cluster/WE material combinations, this process is very slow, leading to low currents and poorly resolved peaks in the CV profiles. In these cases, better insight into the electrochemical behavior of the clusters can be gained by IR SEC experiments, which seem to suffer fewer of these problems.

As a final remark, HNMCCs offer a molecular approach to metal and alloy nanoclusters. Very detailed structural insights (with atomic precision and resolution) can be obtained by SC-XRD, whereas electrochemical and spectroelectrochemical experiments offer detailed information about the electronic status of their metal cores and structural, spectroscopic, and chemical information as the number of electrons is varied.

## 4 Experimental Section

### 4.1 General Procedures

All reactions and sample manipulations were carried out using standard Schlenk techniques under nitrogen and in dried solvents. All of the reagents were commercial products (Aldrich) of the highest purity available and used as received, except [NBu_4_]_2_[Ni_6_(CO)_12_], [NMe_3_CH_2_Ph]_2_[Ni_6_(CO)_12_],^57^ and [Pd(CH_3_CN)_4_][BF_4_]_2_,^58^ which were prepared according to the literature. Analyses of C, H, and N were performed with a Thermo Quest Flash EA 1112NC instrument. Analyses of Ni and Pd were performed by microwave plasma-atomic emission spectrometry on an Agilent 4210 MP-AES instrument. IR spectra were recorded on a PerkinElmer Spectrum One interferometer in CaF_2_ cells. Structural drawings were determined with SCHAKAL99.^59^

**Caution:** CO and Ni(CO)_4_ may be generated during manipulation of these compounds. All of the operations must be carried out under a well-ventilated fume hood.

### 4.2 Synthesis of [NMe_4_]_2_[NMe_3_CH_2_Ph]_4_[Ni_36–*x*_Pd_5+*x*_(CO)_46_]·3CH_3_CN·solv (*x* = 0.41) (**1**^**6–**^)

[Pd(CH_3_CN)_4_][BF_4_]_2_ (0.920 g, 2.07 mmol) was added as a solid in small portions to a solution of [NMe_3_CH_2_Ph]_2_[Ni_6_(CO)_12_] (2.50 g, 2.53 mmol) in thf (80 mL) over a period of 6 h. The resulting mixture was stirred at room temperature for 24 h, and then the solvent removed *in vacuo*. The residue was washed with H_2_O (3 × 20 mL) and extracted with acetone (20 mL). A saturated solution of [NMe_4_]Cl in H_2_O (50 mL) was added to complete the precipitation of the compound. The solid was recovered by filtration, washed with H_2_O (3 × 20 mL), toluene (3 × 20 mL), and thf (20 mL), and extracted with CH_3_CN (20 mL). Crystals of [NMe_4_]_2_[NMe_3_CH_2_Ph]_4_[**1**]·3CH_3_CN·solv suitable for X-ray analyses were obtained by layering *n*-hexane (2 mL) and diisopropyl ether (40 mL) on the CH_3_CN solution (yield of 0.76 g, 37% based on Ni, 41% based on Pd). Anal. Calcd for C_100_H_97_N_9_Ni_35.58_O_46_Pd_5.41_ (4826.21): C, 25.03; H, 2.04; N, 2.63; Ni, 43.00; Pd, 11.95. Found: C, 25.34; H, 2.29; N, 2.31; Ni, 43.28; Pd, 12.15. IR (CH_3_CN, 293 K) ν_CO_: 2005(vs), 1858(s) cm^–1^.

### 4.3 Synthesis of [NBu_4_]_6_[Ni_37–*x*_Pd_7+*x*_(CO)_48_]·6CH_3_CN (*x* = 0.69) (**2**^**6–**^)

[Pd(CH_3_CN)_4_][BF_4_]_2_ (1.25 g, 2.81 mmol) was added as a solid in small portions to a solution of [NBu_4_]_2_[Ni_6_(CO)_12_] (3.50 g, 2.99 mmol) in thf (30 mL) over a period of 6 h. The resulting mixture was stirred at room temperature for 24 h, and then the solvent removed *in vacuo*. The residue was washed with H_2_O (3 × 20 mL), toluene (3 × 20 mL), and thf (20 mL) and extracted with CH_3_CN (20 mL). Crystals of [NBu_4_]_6_[**2**]·6CH_3_CN suitable for X-ray analyses were obtained by layering *n*-hexane (2 mL) and diisopropyl ether (40 mL) on the CH_3_CN solution (yield of 0.89 g, 30% based on Ni, 41% based on Pd). Anal. Calcd for C_156_H_234_N_12_Ni_36.31_O_48_Pd_7.69_ (5995.51): C, 31.40; H, 3.96; N, 2.82; Ni, 35.29; Pd, 13.66. Found: C, 31.16; H, 4.12; N, 2.65; Ni, 34.98; Pd, 13.89. IR (CH_3_CN, 293 K) ν_CO_: 1983(vs), 1838(s) cm^–1^.

### 4.4 Synthesis of [NBu_4_]_5_[HNi_37–*x*_Pd_7+*x*_(CO)_48_]·2CH_3_COCH_3_·solv (*x* = 0.53) (**3**^**5–**^)

[Pd(CH_3_CN)_4_][BF_4_]_2_ (0.426 g, 0.959 mmol) was added as a solid in small portions to a solution of [NBu_4_]_2_[Ni_6_(CO)_12_] (1.17 g, 0.998 mmol) in thf (30 mL) over a period of 6 h. The resulting mixture was stirred at room temperature for 24 h, and then the solvent removed *in vacuo*. The residue was washed with H_2_O (3 × 20 mL), toluene (3 × 20 mL), and thf (20 mL) and extracted with acetone (20 mL). Crystals of [NBu_4_]_5_[**3**]·2CH_3_COCH_3_·solv suitable for X-ray analyses were obtained by layering *n*-hexane (40 mL) on the acetone solution (yield of 0.38 g, 41% based on Ni, 53% based on Pd). Anal. Calcd for C_134_H_192_N_5_Ni_36.47_O_50_Pd_7.53_ (5615.50): C, 28.81; H, 3.47; N, 1.25; Ni, 37.85; Pd, 14.29. Found: C, 28.64; H, 3.26; N, 1.51; Ni, 37.98; Pd, 14.02. IR (nujol, 293 K) ν_CO_: 2004(vs), 1972(sh), 1873(s), 1854(sh) cm^–1^. IR (acetone, 293 K) ν_CO_: 2013(vs), 1875(s) cm^–1^. IR (CH_3_CN, 293 K) ν_CO_: 2006(vs), 1867(s) cm^–1^.

### 4.5 X-ray Crystallographic Study

Crystal data and collection details for [NMe_4_]_2_[NMe_3_CH_2_Ph]_4_[**1**]·3CH_3_CN·solv, [NBu_4_]_6_[**2**]·6CH_3_CN, and [NBu_4_]_5_[**3**]·2CH_3_COCH_3_·solv are listed in Table S1. The diffraction experiments were carried out on a Bruker APEX II diffractometer equipped with a PHOTON100 detector using Mo Kα radiation. Data were corrected for Lorentz polarization and absorption effects (empirical absorption correction SADABS).^60^ Structures were determined by direct methods and refined by full-matrix least squares based on all data using *F*^2^.^61^ Hydrogen atoms were fixed at calculated positions and refined by a riding model. All non-hydrogen atoms were refined with anisotropic displacement parameters, unless otherwise stated. Further details can be found in the Supporting Information.

### 4.6 Electrochemical, Spectroelectrochemical, and Electrochemical Impedance Spectroscopy Measurements

Materials and apparatuses for electrochemistry and IR SEC have been described elsewhere.^14^

A platinum disk (7.39 × 10^–2^ cm^2^) or glassy carbon disk (8.17 × 10^–2^ cm^2^) was used as the working electrode for the voltammetric or EIS experiments, respectively. Their electroactive area was determined using the Randles–Sevcik equation by measuring peak current *i*_p_ at different scan rates for a 1.15 mM FeCp_2_ solution in CH_3_CN/[NBu_4_][PF_6_].^62^ The GC electrode was polished, prior to measurements, according to the following procedure: manual rubbing with a 0.3 μm Al_2_O_3_ slurry in water (eDAQ) for 2 min, sonication in ultrapure water for 10 min, manual rubbing with a 0.05 μm Al_2_O_3_ slurry in water (eDAQ) for 2 min, and sonication in ultrapure water for 10 min. After being polished, the electrodes were rinsed with acetone and air-dried.

EIS spectra were recorded using as *E*_dc_ the *E*°′ of a reversible electrochemical reaction obtained from the voltammetric experiments. *E*_ac_ was set to 0.005 V, and the frequency was scanned between 10000 and 1 Hz. All of the electrochemical experiments were performed using a Palmsens 4 potentiostat.

## Supporting Information Available

Molecular structure of [HNi_37–*x*_Pd_7+*x*_(CO)_48_]^5–^ (*x* = 0.53), supplementary IR SEC figures, and an X-ray crystallographic study (PDF)

## Accession Codes

CCDC 2097872–2097874 contain the supplementary crystallographic data for this paper. These data can be obtained free of charge via www.ccdc.cam.ac.uk/data_request/cif, or by emailing data_request@ccdc.cam.ac.uk, or by contacting The Cambridge Crystallographic Data Centre, 12 Union Road, Cambridge CB2 1EZ, UK; fax: +44 1223 336033.
