# Supplementary material for: Atomically Precise Ni–Pd Alloy Carbonyl Nanoclusters: Synthesis, Total Structure, Electrochemistry, Spectroelectrochemistry, and Electrochemical Impedance Spectroscopy
Source: Inorg Chem. 2021 Oct 21;60(21):16713–25. doi: 10.1021/acs.inorgchem.1c02582 (PMC8564757; doi:10.1021/acs.inorgchem.1c02582)
Supplement: Supplementary file 1 — ic1c02582_si_001.pdf [file ic1c02582_si_001.pdf]

Supporting Information for

Atomically Precise Ni-Pd Alloy Carbonyl Nanoclusters: Synthesis, Total Structure, Electrochemistry, Spectroelectrochemistry and Electrochemical Impedance Spectroscopy

Cristiana Cesari,[a] Tiziana Funaioli,[b]* Beatrice Berti,[a] Cristina Femoni,[a] Maria Carmela Iapalucci,[a] Federico Maria Vivaldi [b] and Stefano Zacchini[a]*

[a] Dipartimento di Chimica Industriale "Toso Montanari", Università di Bologna, Viale Risorgimento 4, 40136 Bologna. Italy. E-mail: stefano.zacchini@unibo.it

[b] Dipartimento di Chimica e Chimica Industriale, Università di Pisa, Via G. Moruzzi 13, 56124, Pisa, Italy. E-mail: tiziana.funaioli@unipi.it

	Page/s
Molecular structure of $[HNi_{37-x}Pd_{7+x}(CO)_{48}]^{5-}$ (x = 0.53)	S2-S3
Experimental IR spectra and CV responses	S4-S13
X-Ray crystallographic data and details	S14-S16
References	S17

Figure S1

(a) The molecular structure of the first independent molecule of $[HNi_{37-x}Pd_{7+x}(CO)_{48}]^{5-}$ (x = 0.53) and (b) its M_{44} metal core. Two views are reported (green, Ni; purple, Pt, yellow, Ni/Pd≈ 42:58; blue, Ni/Pd≈ 88-92:12-8; grey, C; red, O).

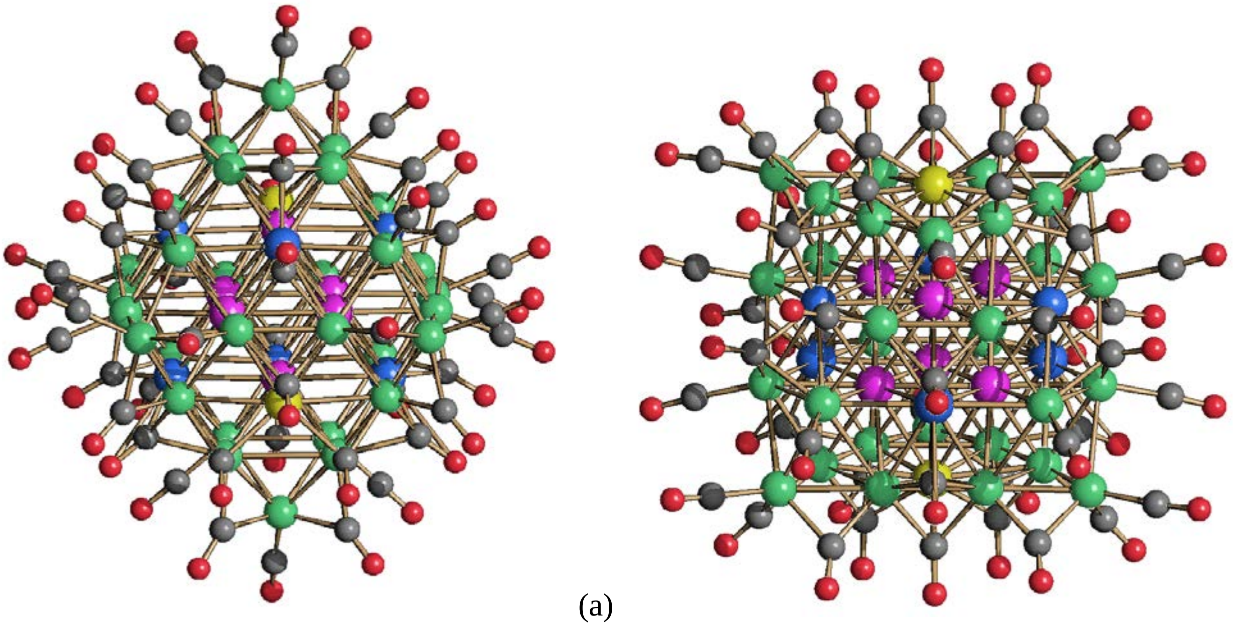

(a)

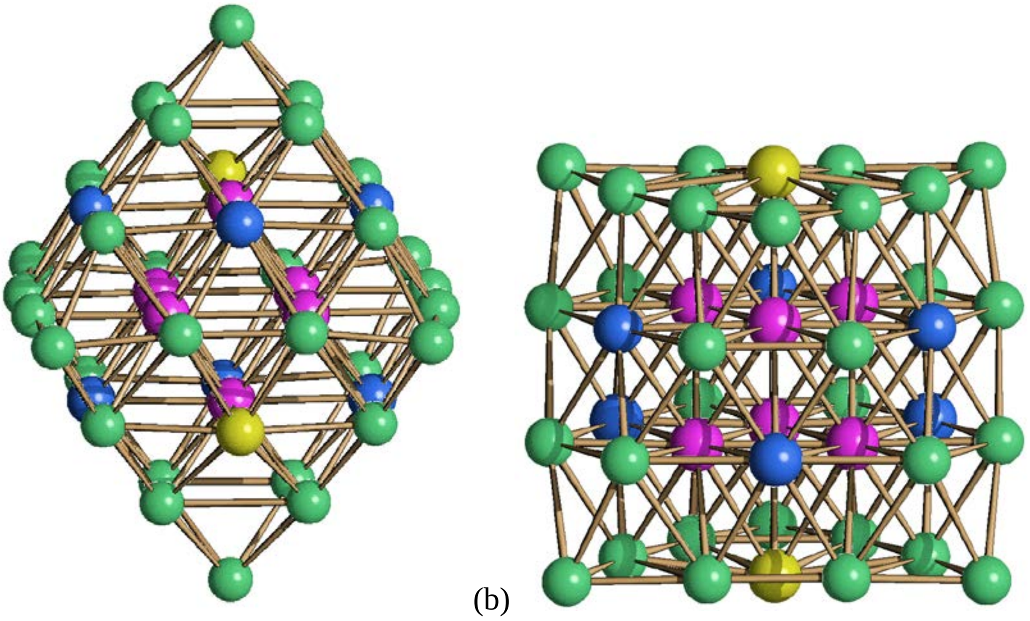

(b)

Figure S2

(a) The molecular structure of the second independent molecule of $[HNi_{37-x}Pd_{7+x}(CO)_{48}]^{5-}$ (x = 0.53) and (b) its M_{44} metal core. Two views are reported (green, Ni; purple, Pt, yellow, Ni/Pd≈ 51:49; blue, Ni/Pd≈ 83:17; grey, C; red, O).

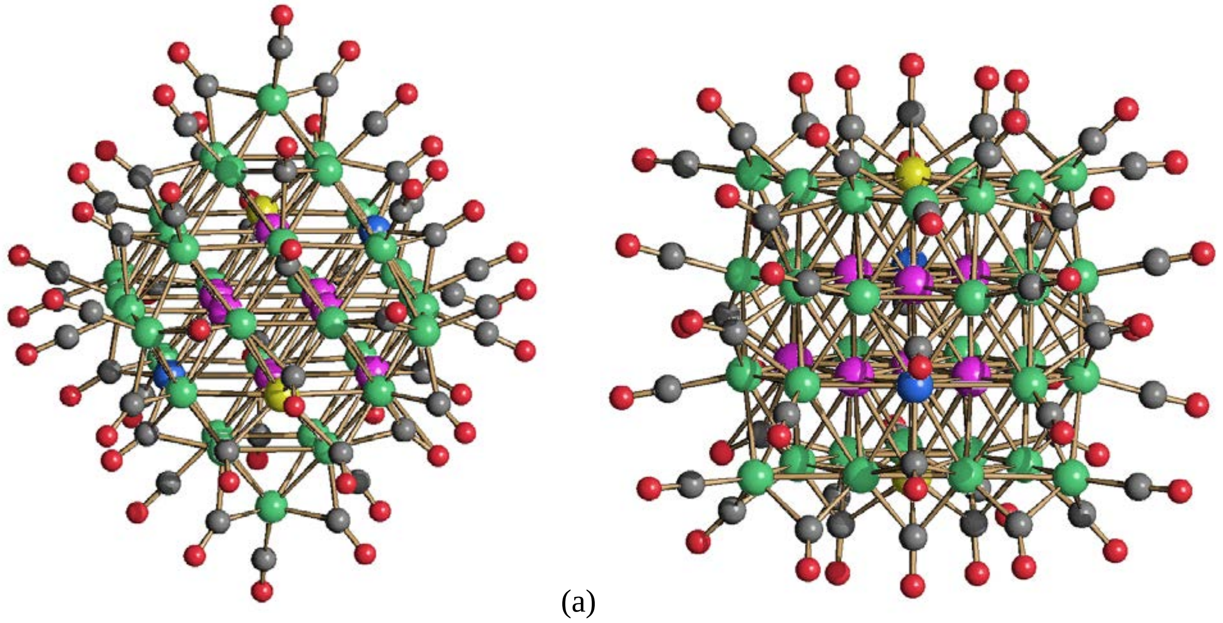

(a)

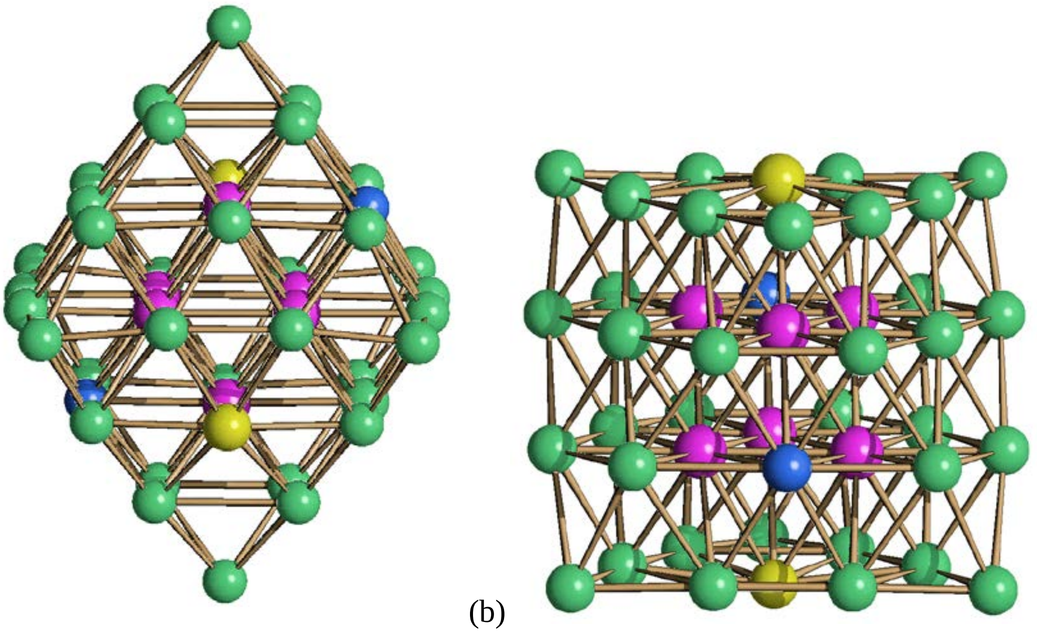

(b)

Figure S3

IR spectral changes of a CH_3CN solution $\mathbf{1}^{6-}$ recorded in an OTTLE cell during the progressive decrease of the potential from –0.54 to –0.84 V *vs*. Ag pseudo-reference electrode (scan rate 1 mV s^{-1}). $[N^nBu_4][PF_6]$ (0.1 mol dm^{-3}) as the supporting electrolyte. The absorptions of the solvent and the supporting electrolyte have been subtracted.

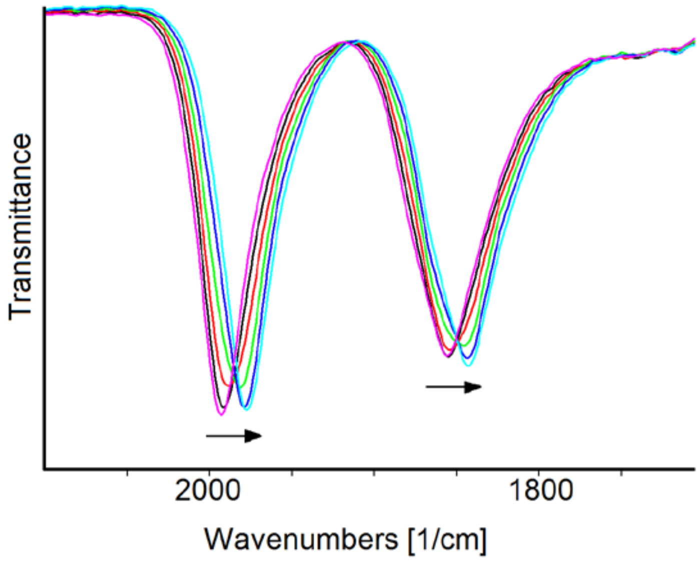

Figure S4

IR spectral changes of a CH_3CN solution $\mathbf{1}^{6-}$ recorded in an OTTLE cell during the progressive decrease of the potential from –0.84 to –1.26 V *vs*. Ag pseudo-reference electrode (scan rate 1 mV s^{-1}). $[N^nBu_4][PF_6]$ (0.1 mol dm^{-3}) as the supporting electrolyte. The absorptions of the solvent and the supporting electrolyte have been subtracted.

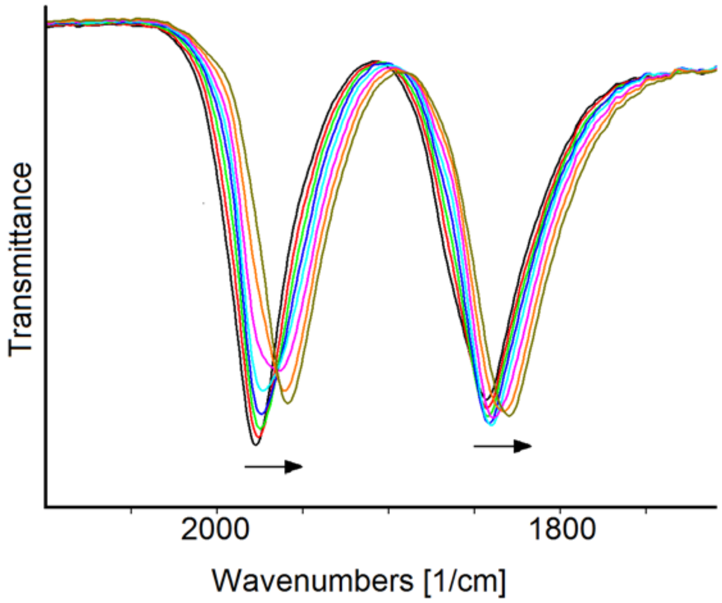

Figure S5

IR spectral changes of a CH_3CN solution **1**$^{6-}$ recorded in an OTTLE cell during the progressive increase of the potential from –1.26 to –1.5 V *vs.* Ag pseudo-reference electrode (scan rate 1 mV s^{-1}). $[N^nBu_4][PF_6]$ (0.1 mol dm^{-3}) as the supporting electrolyte. The absorptions of the solvent and the supporting electrolyte have been subtracted.

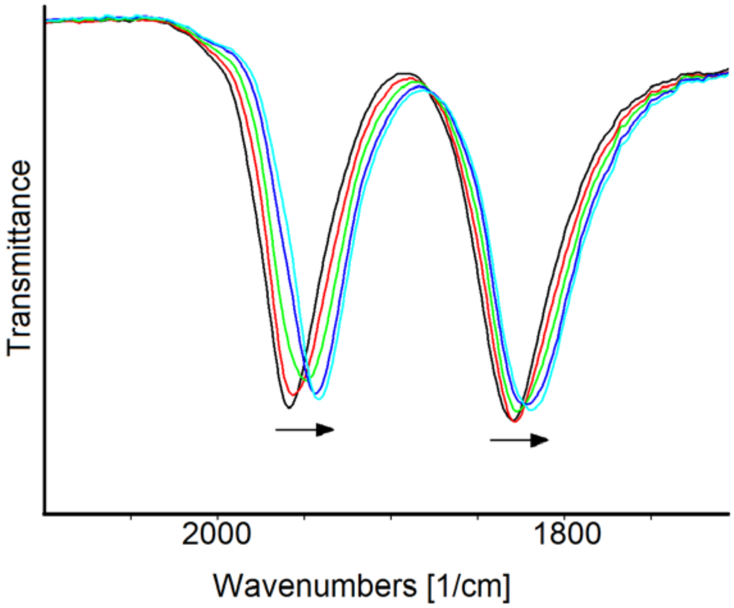

Figure S6

IR spectral changes of a CH_3CN solution **1**$^{6-}$ recorded in an OTTLE cell during the progressive increase of the potential from –0.54 to 0.00 V *vs.* Ag pseudo-reference electrode (scan rate 1 mV s^{-1}). $[N^nBu_4][PF_6]$ (0.1 mol dm^{-3}) as the supporting electrolyte. The absorptions of the solvent and the supporting electrolyte have been subtracted.

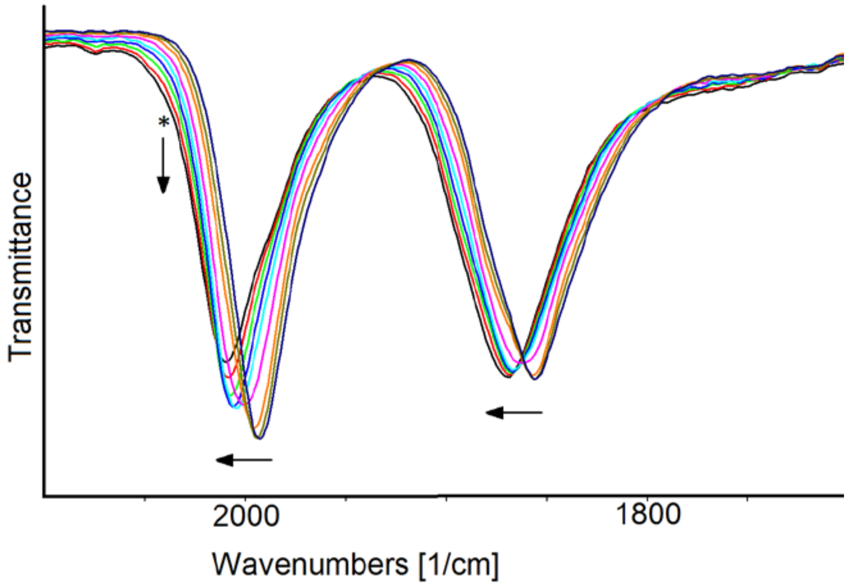

Figure S7

Differential pulse voltammogram recorded at CG electrode in CH_3CN solution of **3**$^{5-}$. [N^nBu_4][PF_6] (0.1 mol dm^{-3}) supporting electrolyte. Starred peaks are attributable to unknown impurities.

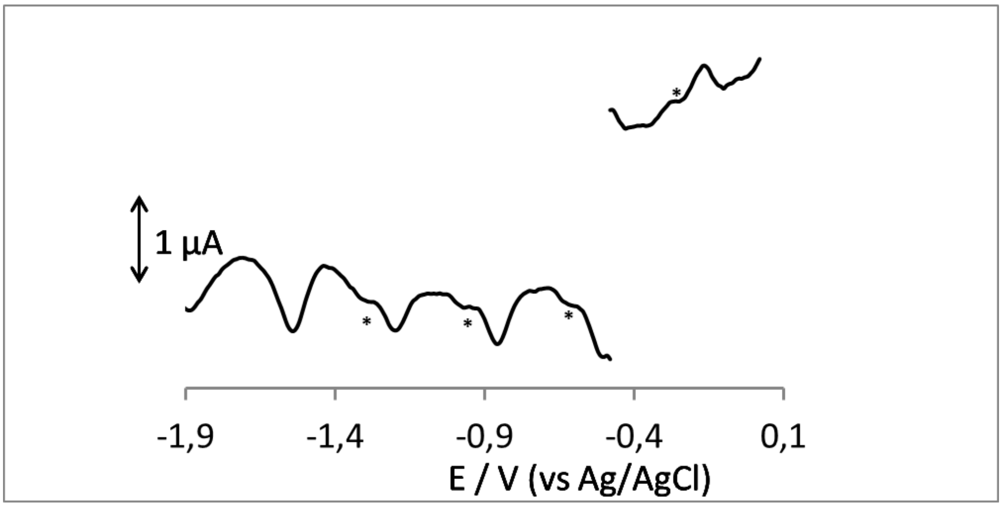

Figure S8

IR spectral changes of a CH_3CN solution **3**$^{5-}$ recorded in an OTTLE cell during the progressive decrease of the potential from –0.21 to –0.76 V *vs*. Ag pseudo-reference electrode (scan rate 1 mV s^{-1}). [N^nBu_4][PF_6] (0.1 mol dm^{-3}) as the supporting electrolyte. The absorptions of the solvent and the supporting electrolyte have been subtracted.

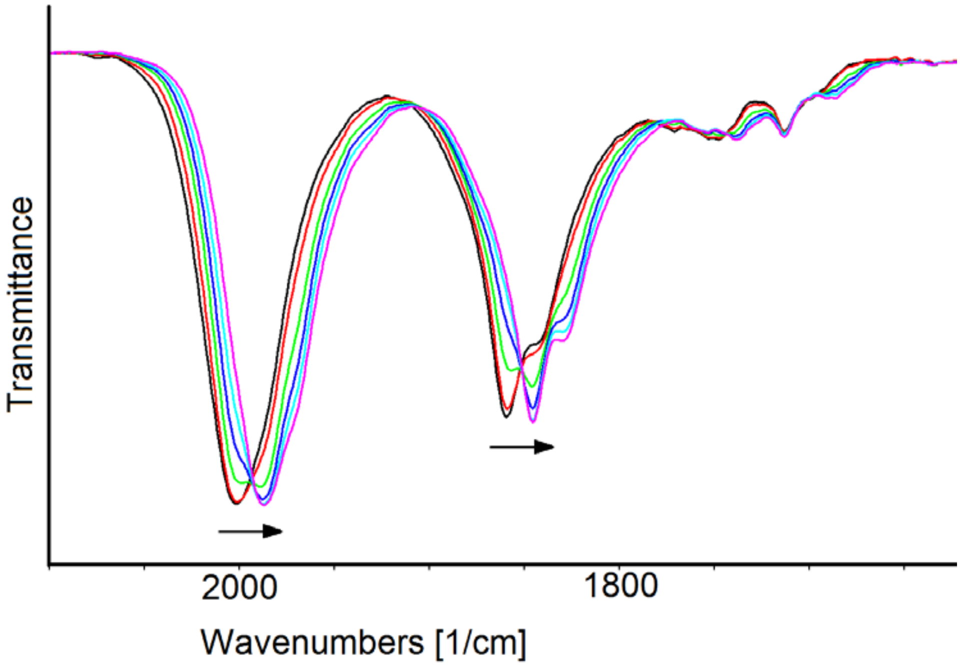

Figure S9

IR spectral changes of a CH_3CN solution **3**$^{5-}$ recorded in an OTTLE cell during the progressive decrease of the potential from –0.76 to –1.20 V *vs*. Ag pseudo-reference electrode (scan rate 1 mV s^{-1}). $[N^nBu_4][PF_6]$ (0.1 mol dm^{-3}) as the supporting electrolyte. The absorptions of the solvent and the supporting electrolyte have been subtracted

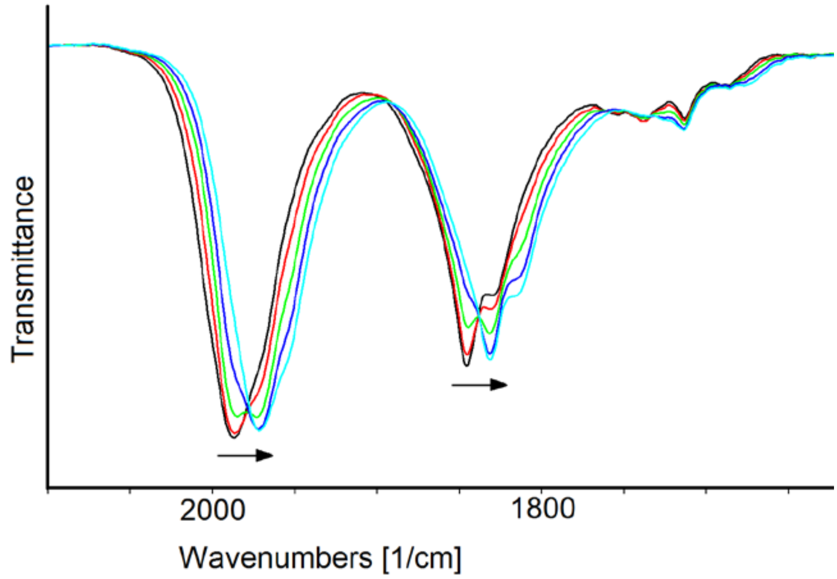

.

Figure S10

IR spectral changes of a CH_3CN solution **3**$^{5-}$ recorded in an OTTLE cell during the progressive decrease of the potential from –1.20 to –1.60 V *vs*. Ag pseudo-reference electrode (scan rate 1 mV s^{-1}). $[N^nBu_4][PF_6]$ (0.1 mol dm^{-3}) as the supporting electrolyte. The absorptions of the solvent and the supporting electrolyte have been subtracted.

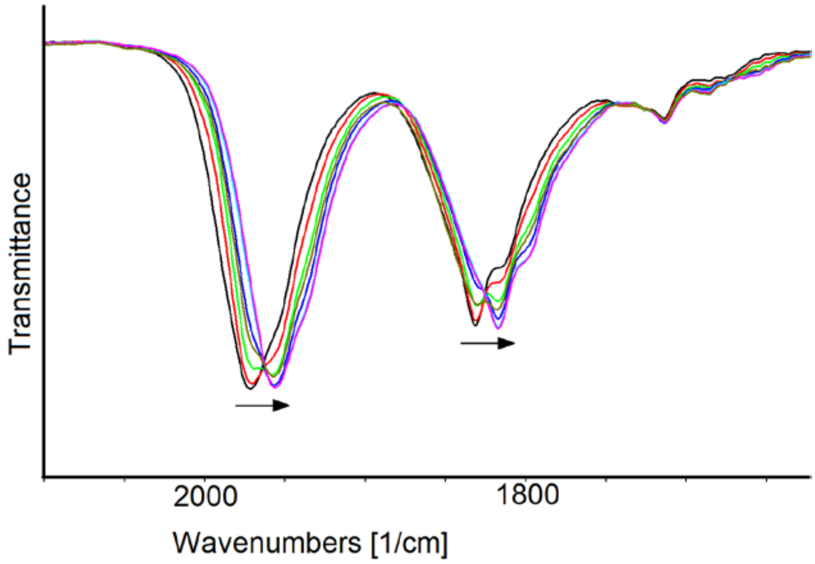

Figure S11

IR spectral changes of a CH_3CN solution $\mathbf{3}^{5-}$ recorded in an OTTLE cell during the progressive decrease of the potential from –1.60 to –2.00 V *vs*. Ag pseudo-reference electrode (scan rate 1 mV s^{-1}). $[N^nBu_4][PF_6]$ (0.1 mol dm^{-3}) as the supporting electrolyte. The absorptions of the solvent and the supporting electrolyte have been subtracted.

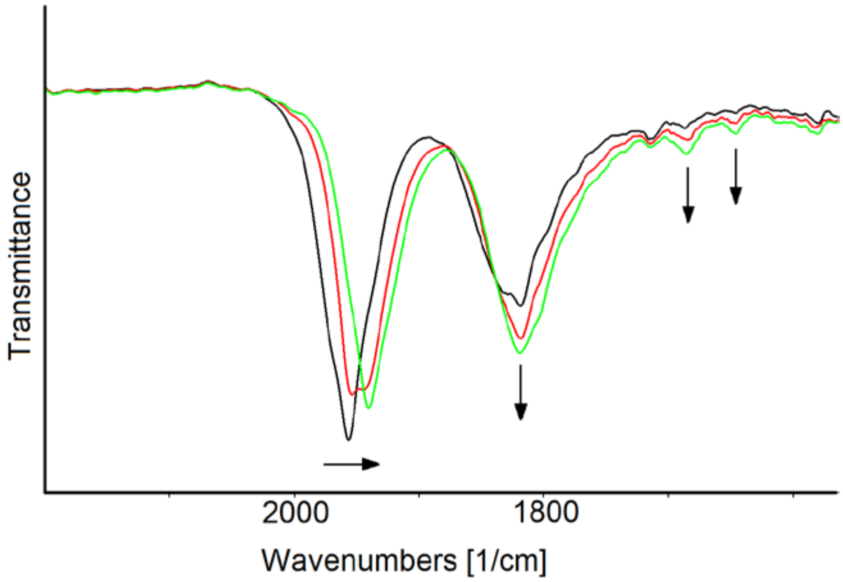

Figure S12

IR spectral changes of a CH_3CN solution $\mathbf{3}^{5-}$ recorded in an OTTLE cell during the progressive decrease of the potential from –2.00 to –2.20 V *vs*. Ag pseudo-reference electrode (scan rate 1 mV s^{-1}). $[N^nBu_4][PF_6]$ (0.1 mol dm^{-3}) as the supporting electrolyte. The absorptions of the solvent and the supporting electrolyte have been subtracted.

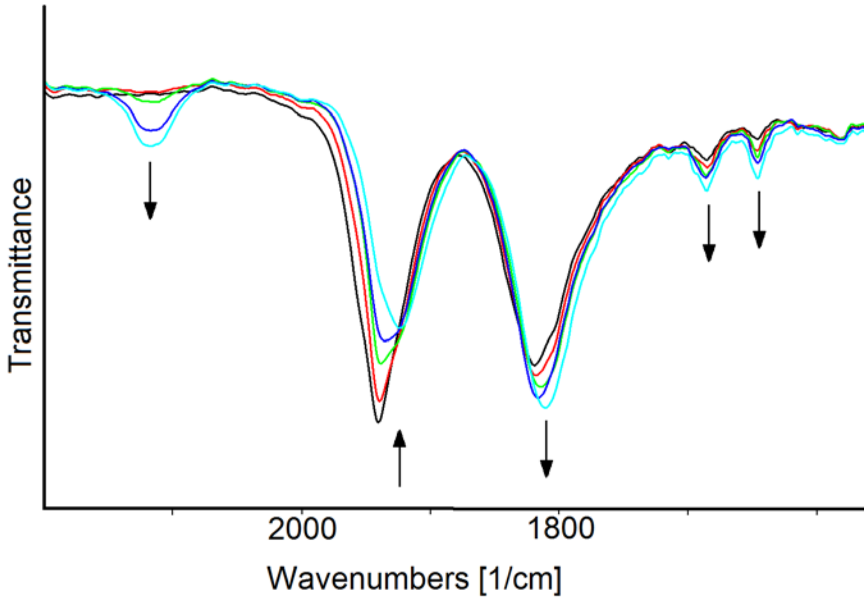

Figure S13

IR spectral changes of a CH_3CN solution **3**$^{5-}$ recorded in an OTTLE cell during the progressive increase of the potential from –0.21 to 0.10 V *vs.* Ag pseudo-reference electrode (scan rate 1 mV s^{-1}). $[N^nBu_4][PF_6]$ (0.1 mol dm^{-3}) as the supporting electrolyte. The absorptions of the solvent and the supporting electrolyte have been subtracted.

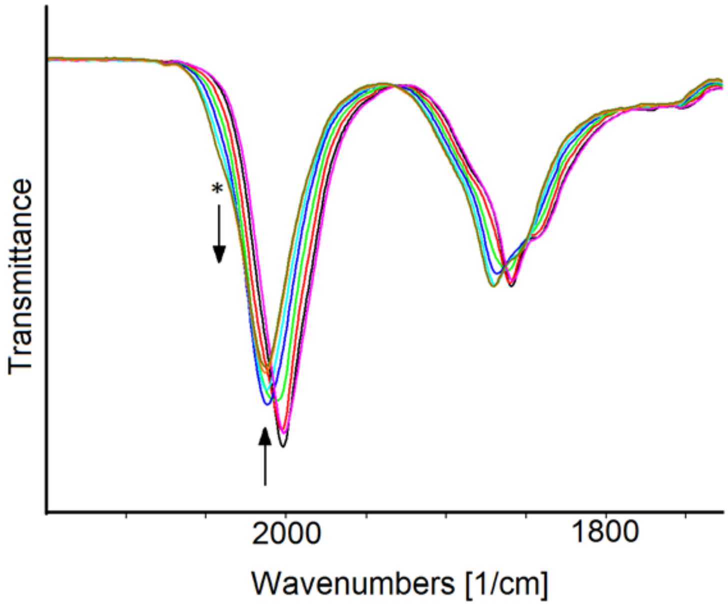

Figure S14

IR spectra of a CH_3CN solution **3**$^{5-}$ recorded in an OTTLE cell before (black line) and after (red line) the cyclic swept of the potential from −0.21 to 0.10 V *vs.* Ag pseudo-reference electrode (scan rate 1 mV s^{-1}). $[N^nBu_4][PF_6]$ (0.1 mol dm^{-3}) as the supporting electrolyte. The absorptions of the solvent and the supporting electrolyte have been subtracted

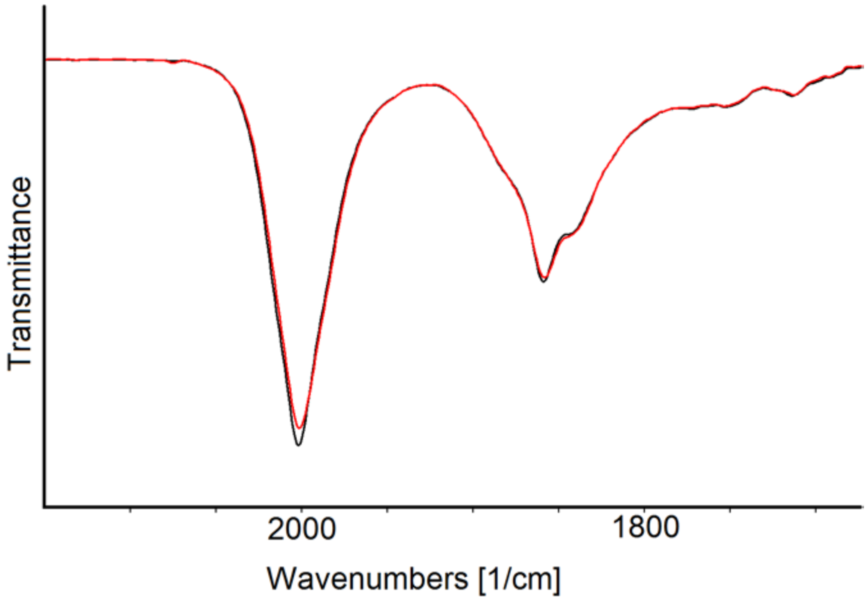

Figure S15

CV response of $\mathbf{3}^{5-}$ (red line) and $\mathbf{2}^{6-}$ (blue line) at a GC electrode in CH_3CN solution of $[N^nBu_4][PF_6]$ (0.1 mol dm^{-3}) supporting electrolyte. Scan rate: 0.2 V s^{-1}.

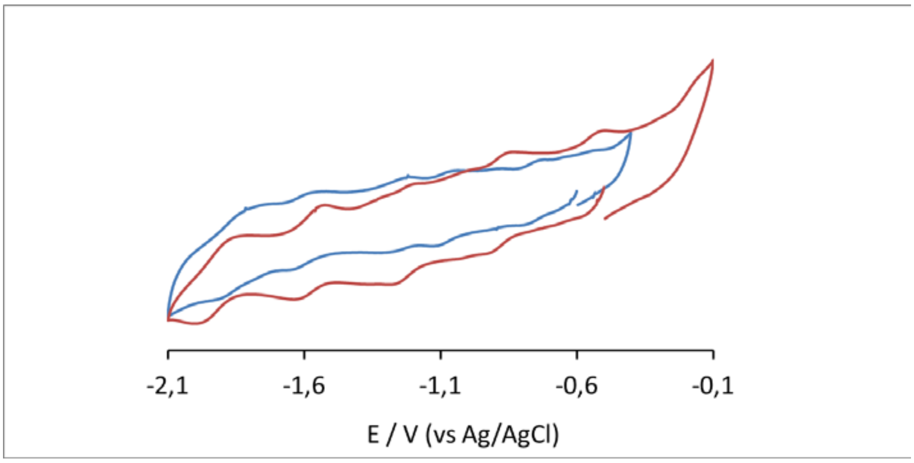

Figure S16

IR spectral changes of a CH_3CN solution $\mathbf{2}^{6-}$ recorded in an OTTLE cell during the progressive increase of the potential from −0.6 to −0.3 V *vs.* Ag pseudo-reference electrode (scan rate 1 mV s^{-1}). $[N^nBu_4][PF_6]$ (0.1 mol dm^{-3}) as the supporting electrolyte. The absorptions of the solvent and the supporting electrolyte have been subtracted. A reference spectrum, collected before the application of an oxidant potential, is used to calculate the differential absorbance spectra reported in the top of the figure.

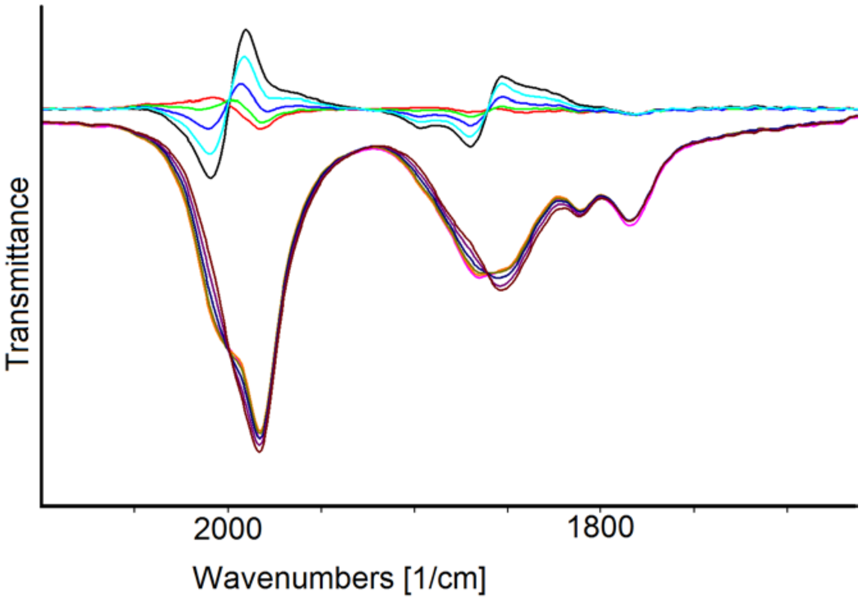

Figure S17

IR spectral changes of a CH_3CN solution **2**$^{6-}$ recorded in an OTTLE cell during the progressive decrease of the potential from −0.6 to −1.2 V *vs.* Ag pseudo-reference electrode (scan rate 1 mV s^{-1}). [N^{n}Bu$_4$][PF$_6$] (0.1 mol dm^{-3}) as the supporting electrolyte. The absorptions of the solvent and the supporting electrolyte have been subtracted. A reference spectrum, collected before the application of an reducing potential, is used to calculate the differential absorbance spectra reported in the top of the figure.

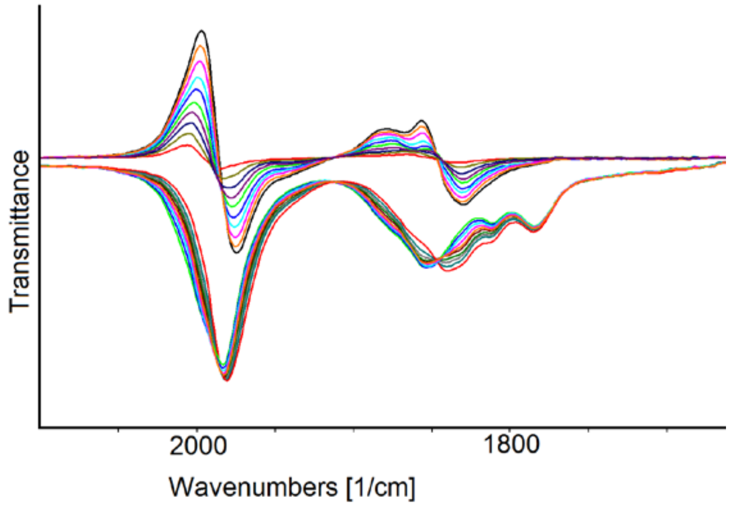

Figure S18

IR spectral changes of a CH_3CN solution **2**$^{6-}$ recorded in an OTTLE cell during the progressive decrease of the potential from −1.2 to −1.5 V *vs.* Ag pseudo-reference electrode (scan rate 1 mV s^{-1}). [N^{n}Bu$_4$][PF$_6$] (0.1 mol dm^{-3}) as the supporting electrolyte. The absorptions of the solvent and the supporting electrolyte have been subtracted. To calculate the differential absorbance spectra reported in the top of the figure, as reference spectrum, was used the first one of the series, collected at the potential of −1.2 V.

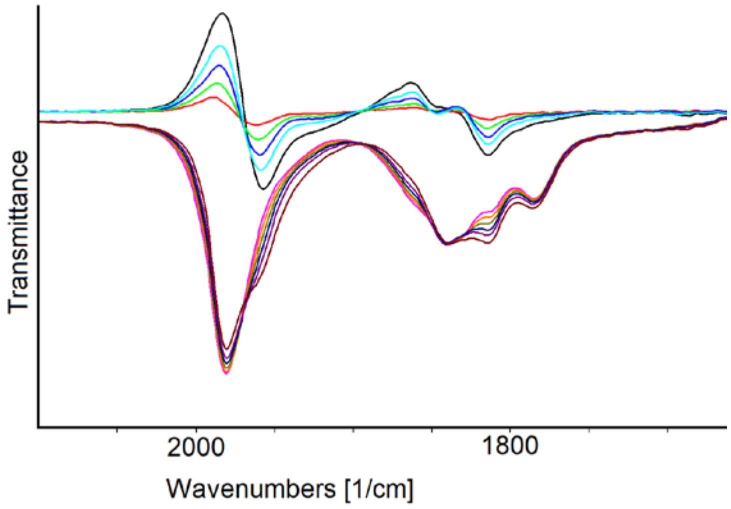

Figure S19

IR spectral changes of a CH_3CN solution **2**$^{6-}$ recorded in an OTTLE cell during the progressive decrease of the potential from −1.5 to −1.8 V *vs.* Ag pseudo-reference electrode (scan rate 1 mV s^{-1}). [N^nBu_4][PF_6] (0.1 mol dm^{-3}) as the supporting electrolyte. The absorptions of the solvent and the supporting electrolyte have been subtracted. To calculate the differential absorbance spectra reported in the top of the figure, as reference spectrum, was used the first one of the series, collected at the potential of −1.5 V.

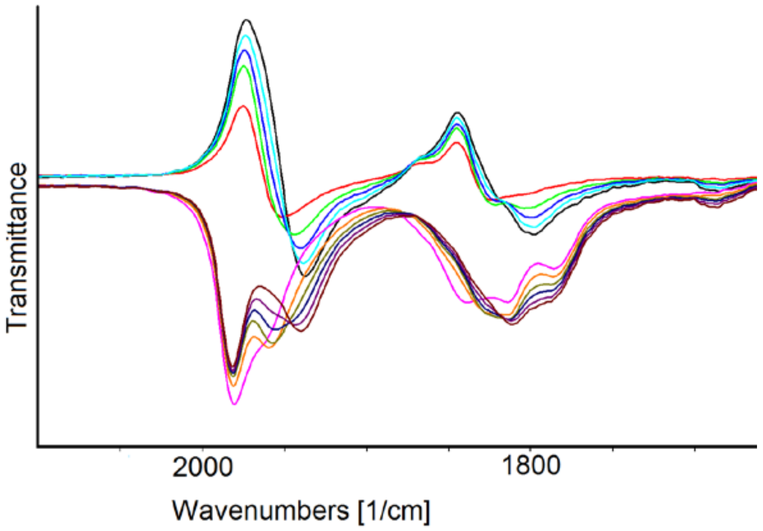

Figure S20

IR spectra of a CH_3CN solution **2**$^{6-}$ recorded in an OTTLE cell before (black line) and after (red line) the cyclic swept of the potential from −0.3 to −1.9 V *vs.* Ag pseudo-reference electrode (scan rate 1 mV s^{-1}). [N^nBu_4][PF_6] (0.1 mol dm^{-3}) as the supporting electrolyte. The absorptions of the solvent and the supporting electrolyte have been subtracted.

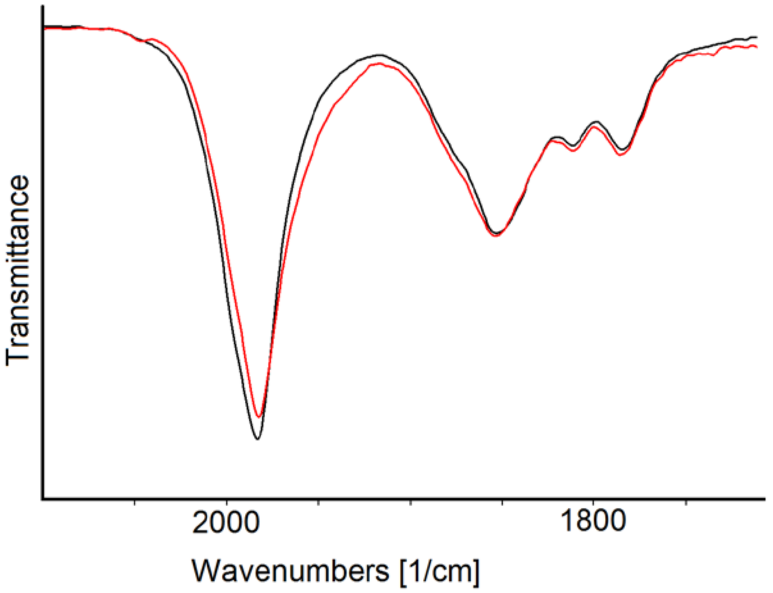

Figure S21

CV response of $[Pt_{19}(CO)_{22}]^{4-}$ at a GC (blue line) and at a Pt (red line) electrodes in CH_3CN solution of $[N^nBu_4][PF_6]$ (0.1 mol dm^{-3}) supporting electrolyte. Scan rate: 0.1 V s^{-1}.

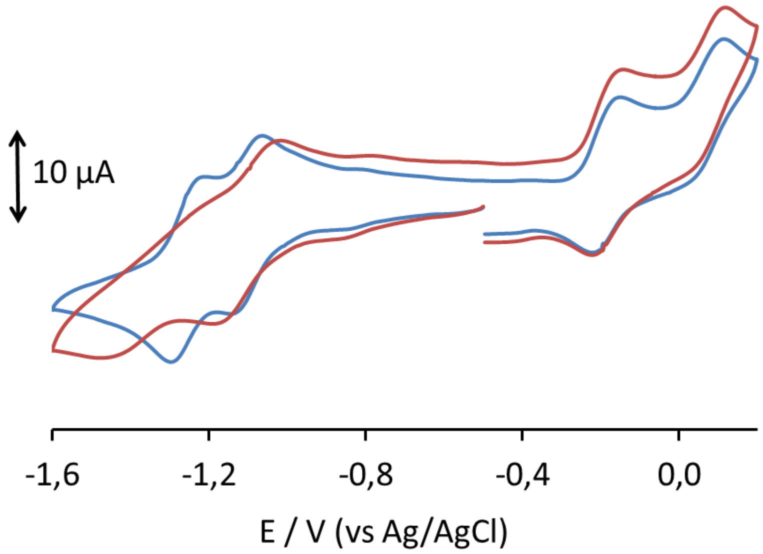

X-ray Crystallographic Study.

Crystal data and collection details for $[NBu_4]_5[HNi_{37-x}Pd_{7+x}(CO)_{48}]\cdot 2CH_3COCH_3\cdot$solv (x = 0.53), $[NBu_4]_6[Ni_{37-x}Pd_{7+x}(CO)_{48}]\cdot 6CH_3CN$ (x = 0.69), $[NMe_4]_2[NMe_3CH_2Ph]_4[Ni_{36-x}Pd_{5+x}(CO)_{46}]\cdot 3CH_3CN\cdot$solv (x = 0.41), are reported in Table S1. The diffraction experiments were carried out on a Bruker APEX II diffractometer equipped with a PHOTON100 detector using Mo–Kα radiation. Data were corrected for Lorentz polarization and absorption effects (empirical absorption correction SADABS).[1] Structures were solved by direct methods and refined by full-matrix least-squares based on all data using F^2.[2] Hydrogen atoms were fixed at calculated positions and refined by a riding model. All non-hydrogen atoms were refined with anisotropic displacement parameters, unless otherwise stated.

$[NBu_4]_5[HNi_{37-x}Pd_{7+x}(CO)_{48}]\cdot 2CH_3COCH_3\cdot$solv (x = 0.53): The asymmetric unit of the unit cell contains two halves of two cluster anions (located on inversion centres), five $[NBu_4]^+$ cations and two CH_3COCH_3 molecules located on general positions. One CH_3COCH_3 molecule is disordered and, thus, it has been split into two positions and refined with one occupancy parameter per disordered group. The unit cell contains an additional total potential solvent accessible void of 478 Å^3 (*ca*. 5% of the Cell Volume), which is likely to be occupied by further highly disordered CH_3COCH_3 molecules. These voids have been treated using the SQUEEZE routine of PLATON.[3] The positions occupied by M(2), M(8), M(11), M(18), M(31) and M(34) are disordered Ni/Pd. These have been refined applying dummy atoms constraints (EADP and EXYZ lines in SHLEXL) resulting in the following refined occupancy factors: M(2) = 0.91(2) Ni and 0.09(2) Pd; M(8) = 0.42(2) Ni and 0.58(2) Pd; M(11) = 0.88(2) Ni and 0.12(2) Pd; M(18) = 0.92(2) Ni and 0.08(2) Pd; M(31) = 0.83(2) Ni and 0.17(2) Pd; M(34) = 0.51(2) Ni and 0.49(2) Pd. All C, O and N atoms have been restrained to isotropic like behaviour (ISOR line in SHELXL, s.u. 0.01). Similar *U* parameter restraints have been applied to the $[NBu_4]^+$ cations and CH_3COCH_3 molecules (SIMU line in SHELXL, s.u. 0.02). Restraints to bond distances were applied as follow (s.u. 0.02): 1.47 Å for C–N and 1.53 Å for C–C in $[NBu_4]^+$; 1.24 Å for C–O and 1.51 Å for C–C in CH_3COCH_3.

$[NBu_4]_6[Ni_{37-x}Pd_{7+x}(CO)_{48}]\cdot 6CH_3CN$ (x = 0.69): The asymmetric unit of the unit cell contains one sixth of a cluster anion (located on $\bar{3}$), one $[NBu_4]^+$ cation and one CH_3CN molecule located on general positions. The CH_3CN molecule is disordered and, thus, it has been split into two positions and refined with one occupancy parameter per disordered group. The positions occupied by M(2) and M(3) are disordered Ni/Pd. These have been refined applying dummy atoms constraints (EADP and EXYZ lines in SHLEXL) resulting in the following refined occupancy factors: M(2) = 0.328(17) Ni and 0.672(17) Pd; M(3) = 0.906(11) Ni and 0.094(11) Pd. The atoms of the $[NBu_4]^+$ cation have been restrained to have similar *U* parameters (SIMU line in SHELXL, s.u. 0.01) and

isotropic like behavior (ISOR line in SHELXL, s.u. 0.01). Restraints to bond distances were applied as follow (s.u. 0.01): 1.47 Å for C–N and 1.53 Å for C–C in $[NBu_4]^+$.

$[NMe_4]_2[NMe_3CH_2Ph]_4[Ni_{36-x}Pd_{5+x}(CO)_{46}]\cdot 3CH_3CN\cdot solv$ (x = 0.41): The asymmetric unit of the unit cell contains one third of a cluster anion (located on 3), one $[NMe_3CH_2Ph]^+$ cation (located on a general position in proximity of 3) disordered over two positions with occupancy factors 0.66667 and 0.33333, one third of a disordered $[NMe_3CH_2Ph]^+/[NMe_4]^+$ cation located on 3, two thirds of a $[NMe_4]^+$ cation disordered over three equally populated symmetry related positions (by 6_3), and one CH_3CN molecule located on a general position. The unit cell contains an additional total potential solvent accessible void of 337 $Å^3$ (*ca*. 5% of the Cell Volume), which is likely to be occupied by further highly disordered CH_3CN molecules. These voids have been treated using the SQUEEZE routine of PLATON.[3] The position occupied by M(3) is disordered Ni/Pd. This has been refined applying dummy atoms constraints (EADP and EXYZ lines in SHLEXL) resulting in the following refined occupancy factor: M(3) = 0.526(18) Ni and 0.474(18) Pd. The crystals appear to be racemically twinned with refined batch factor 0.08(4). Similar *U* parameter restraints have been applied to the $[NMe_3CH_2Ph]^+$ and $[NMe_4]^+$ cations and CH_3CN molecules (SIMU line in SHELXL, s.u. 0.02). The atoms of the Ph-rings of $[NMe_3CH_2Ph]^+$ have been constrained to fit regular hexagons (AFIX 66 line in SHELXL). Restraints to bond distances were applied as follow (s.u. 0.02): 1.47 Å for C–N and 1.51 Å for C–C in $[NMe_3CH_2Ph]^+$; 1.47 Å for C–N in $[NMe_4]^+$; 1.14 Å for C–N and 1.47 Å for C–C in CH_3CN.

Table S1

Crystal data and experimental details for $[NBu_4]_5[HNi_{37-x}Pd_{7+x}(CO)_{48}]\cdot 2CH_3COCH_3\cdot$solv (x = 0.53), $[NBu_4]_6[Ni_{37-x}Pd_{7+x}(CO)_{48}]\cdot 6CH_3CN$ (x = 0.69), $[NMe_4]_2[NMe_3CH_2Ph]_4[Ni_{36-x}Pd_{5+x}(CO)_{46}]\cdot 3CH_3CN\cdot$solv (x = 0.41).

	**$[NBu_4]_5[HNi_{37-x}Pd_{7+x}(CO)_{48}]\cdot 2CH_3COCH_3\cdot$solv (x = 0.53)**	**$[NBu_4]_6[Ni_{37-x}Pd_{7+x}(CO)_{48}]\cdot 6CH_3CN$ (x = 0.69)**	**$[NMe_4]_2[NMe_3CH_2Ph]_4[Ni_{36-x}Pd_{5+x}(CO)_{46}]\cdot 3CH_3CN\cdot$solv (x = 0.41)**
Formula	$C_{134}H_{192}N_5Ni_{36.47}O_{50}Pd_{7.53}$	$C_{156}H_{234}N_{12}Ni_{36.31}O_{48}Pd_{7.69}$	$C_{100}H_{97}N_9Ni_{35.58}O_{46}Pd_{5.41}$
Fw	5615.50	5995.51	4826.21
T, K	100(2)	100(2)	100(2)
λ, Å	0.71073	0.71073	0.71073
Crystal system	Triclinic	Trigonal	Hexagonal
Space Group	$P\bar{1}$	$R\bar{3}$	$P6_3$
a, Å	19.007(3)	29.0227(14)	17.7999(19)
b, Å	20.225(3)	29.0227(14)	17.7999(19)
c, Å	23.265(3)	20.1792(11)	26.648(3)
α, °	90.000(3)	90	90
β, °	88.197(4)	90	90
γ, °	88.739(4)	120	120
Cell Volume, $Å^3$	8937(2)	14720.1(16)	7311.9(17)
Z	2	3	2
D_c, g cm^{-3}	2.087	2.029	2.192
μ, mm^{-1}	4.540	4.141	5.173
F(000)	5597	9025	4747
Crystal size, mm	0.21×0.18×0.12	0.16×0.13×0.11	0.18×0.16×0.12
θ limits, °	0.876–25.000	1.909–25.097	1.526–25.990
Index ranges	$-22 \le h \le 22$<br>$-24 \le k \le 24$<br>$-27 \le l \le 27$	$-34 \le h \le 34$<br>$-34 \le k \le 34$<br>$-24 \le l \le 24$	$-21 \le h \le 21$<br>$-21 \le k \le 21$<br>$-32 \le l \le 32$
Reflections collected	88629	56876	88935
Independent reflections	30951 [R_{int} = 0.1020]	5781 [R_{int} = 0.0707]	9511 [R_{int} = 0.0957]
Completeness to θ max	98.3%	99.0%	99.8%
Data / restraints / parameters	30951 / 1535 / 1996	5781 / 272 / 417	9511 / 451 / 663
Goodness on fit on F^2	1.040	1.060	1.078
R_1 (I > 2σ(I))	0.1236	0.0791	0.0588
wR_2 (all data)	0.3279	0.1757	0.1347
Largest diff. peak and hole, e $Å^{-3}$	4.856 / –2.438	2.883 / –1.175	1.614 / –1.184

References

1. Sheldrick, G. M. SADABS-2008/1 - Bruker AXS Area Detector Scaling and Absorption Correction; Bruker AXS: Madison, WI, 2008.
2. Sheldrick, G. M. Crystal Structure Refinement with SHELXL. *Acta Crystallogr., Sect. C: Struct. Chem.* **2015**, *C71*, 3-8.
3. (a) Spek, A. L. Single-crystal structure validation with the program *PLATON*. *J. Appl. Cryst.* **2003**, *36*, 7-13. (b) Spek, A. L. Structure validation in chemical crystallography. *Acta Cryst.* **2009**, *D65*, 148-155.
